# Organic-Inorganic Hybrid Polymers as Adsorbents for Removal of Heavy Metal Ions from Solutions: A Review

**DOI:** 10.3390/ma7020673

**Published:** 2014-01-27

**Authors:** Babak Samiey, Chil-Hung Cheng, Jiangning Wu

**Affiliations:** 1 Department of Chemistry, Faculty of Science, Lorestan University, Khoramabad 68137-17133, Iran; 2 Department of Chemical Engineering, Ryerson University, Toronto, ON M5B 2K3, Canada

**Keywords:** organic-inorganic hybrid polymer, heavy metal ion, wastewater treatment, adsorption, sol-gel method, self-assembly process, nanobuilding blocks, interpenetrating networks

## Abstract

Over the past decades, organic-inorganic hybrid polymers have been applied in different fields, including the adsorption of pollutants from wastewater and solid-state separations. In this review, firstly, these compounds are classified. These compounds are prepared by sol-gel method, self-assembly process (mesopores), assembling of nanobuilding blocks (e.g., layered or core-shell compounds) and as interpenetrating networks and hierarchically structures. Lastly, the adsorption characteristics of heavy metals of these materials, including different kinds of functional groups, selectivity of them for heavy metals, effect of pH and synthesis conditions on adsorption capacity, are studied.

## 1. Introduction

A wide variety of toxic inorganic and organic chemicals are discharged into the environment as industrial wastes, causing serious water, air, and soil pollution. Water pollution caused by toxic heavy metal ions has become a serious environmental problem. Heavy metals (such as Pt, Pd, Ag, Cu, Cd, Pb, Hg, Ni, Co, Zn, *etc*.) are natural constituents of the earth crust and present in the environment as a result of weathering and erosion of parent rocks [1]. In addition to natural sources, they are introduced in ecosystems through wastewaters originating from anthropogenic sources such as chemical manufacturing, metal finishing, welding, alloys manufacturing, painting, mining, extractive metallurgy, plating, tannery and battery industry and using metal-containing fertilizers and pesticides [2].

These toxic metal ions, even at low concentrations, have deteriorated water resources and drinking water and easily accumulated in the human body throughout the food chain, causing a variety of diseases and disorders [3]. So, it is necessary to remove these metal ions from industrial effluents for their subsequent safe disposal.

The removal of heavy metal ions from wastewaters has been a subject of extensive industrial research. At the same time, some of them (e.g., Pt and Au) are precious and can be recycled and reused for extensive applications [4,5]. The recovery of heavy or valuable metals from water or wastewaters can often result in considerable cost savings and have both ecological and economic benefits.

Different methods, such as precipitation [6], solvent extraction [7] chemical and electrochemical techniques [8], ion-exchange methods [9] ultrafiltration [10] and reverse osmosis [11,12], flotation [13] and coagulation [14] have been developed for the removal of toxic metal ions from industrial effluents and wastewaters. However, most of these processes are unacceptable, owing to the disposal of sludge, their high cost, low efficiency and inapplicability to a wide range of pollutants [15].

Adsorption is a well-known separation method and recognized as one of efficient and economic methods for water decontamination applications. In addition, owing to the reversible nature of most adsorption processes, the adsorbents can be regenerated by suitable desorption processes for multiple uses [16], and many desorption processes are of low maintenance cost, high efficiency, and ease of operation [17].

However, the major problem in this field is to select novel types of adsorbents. A number of adsorbents such as activated carbon [18], zeolites [19,20], clays [21,22] and agricultural residues [23–25] have been used for the removal of heavy metal ions. However, the major disadvantages of these adsorbents are their low adsorption capacities, their relatively weak interactions with metallic ions and difficulties of separation and regeneration of some of them from water. Ion-exchange resins can remove metal ions substantially; however, they have low selectivities and show a high degree of swelling combined with poor mechanical stability [26].

To overcome these limitations, more recently, promising organic-inorganic hybrid polymers have been used for the removal of toxic species from wastewater [27–35]. In these compounds, the functional variation of organic materials is combined with advantages of a thermally stable and robust inorganic substrate, resulting in strong binding affinities toward selected metal ions and relatively high metal ion adsorption capacities. Functionalized hybrid polymeric materials as adsorbent are regarded as one of the most effective techniques because metal ions can be chemically bonded by the organic-inorganic polymer hybrids. These kinds of materials often present the best properties of each of its components in a synergic way and have high performances of physical, chemical and mechanical properties [36].

Organic-inorganic hybrid polymeric materials are currently intensely studied [37] for their efficient applications. The intrinsic multifunctional character of these materials makes them potentially useful in multiple fields. Different forms of organic-inorganic hybrids have been intensively studied due to their interesting properties resulting in a number of applications such as electroanalytical applications [38], their extensive applications as membranes for ultra-and nanofiltration [39,40], superhydrophobic surfaces [41,42], highly transparent films [43,44], pH sensitive composites [45], solar cells [46,47], electrolyte [48], molecular shuttles [49], semiconductors [50], gas separation [51,52], catalysts [53], biosensors [54], drug delivery systems [55], coatings for corrosion protection [56], adsorbents of toxic compounds [27–35], Fire retardant polymers [57], biomaterials for osteo-reconstructive surgery [58], materials for telecommunications or information displays [59], *etc*.

This review describes classifications and synthesis methods of organic-inorganic hybrid polymers and particular attention will be focused on application of them for the adsorption of heavy metals from water as well as their performances and mechanisms.

## 2. Classification and Synthesis of Organic-Inorganic Hybrid Polymers

Organic-inorganic hybrid polymers are classes of materials whose structure includes both organic and inorganic units that interact with each other at the molecular level. These materials are divided into two classes on the basis of interaction between organic and inorganic components. In class I, organic and inorganic are embedded and there are weak interactions, such as hydrogen bonding, van der Waals, *π*–*π* or weak electrostatic interactions between them and in class II, these two components are bonded together through strong covalent or coordinative bonds [37]. Organic-inorganic hybrid polymers are obtained through (1) sol-gel process; (2) self-assembly process; (3) assembling or dispersion of nanobuilding blocks; (4) **hierarchical structures** [37] and interpenetrating networks [60].

### 2.1. Sol-Gel Process

**Scheme 1.**
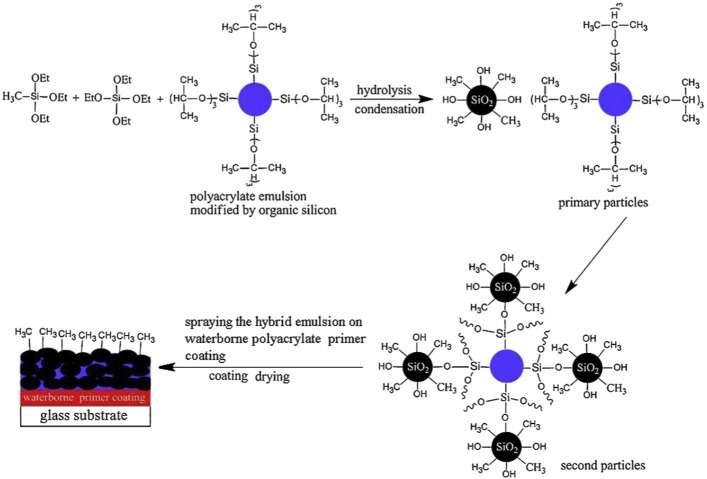
Schematic superhydrophobic surfaces prepared through the sol-gel derived organic-inorganic hybrid emulsion. Reprinted with permission from [41]. Copyright 2011 Elsevier.

In 1846, Ebelmanl and Graham reported that the hydrolysis of tetraethylorthosilicate (TEOS), under acidic conditions produced SiO_2_ in the form of fibres [61,62]. In 1950s, Brady *et al*. [63] produced a number of phenylsilsesquioxane-alkylsilsesquioxane copolymers that were the first successful commercial organic-inorganic hybrid polymers. The sol-gel process is a cheap and low-temperature method for producing transparent and homogenous solid materials with high purity from small molecules and for controlling the chemical composition of products. Compounds produced by the sol-gel process have many applications in superhydrophobic surfaces [41] (shown in Scheme 1), electrolyte [48], biosensors [54], corrosion protection [56], *etc*.

**Scheme 2.**
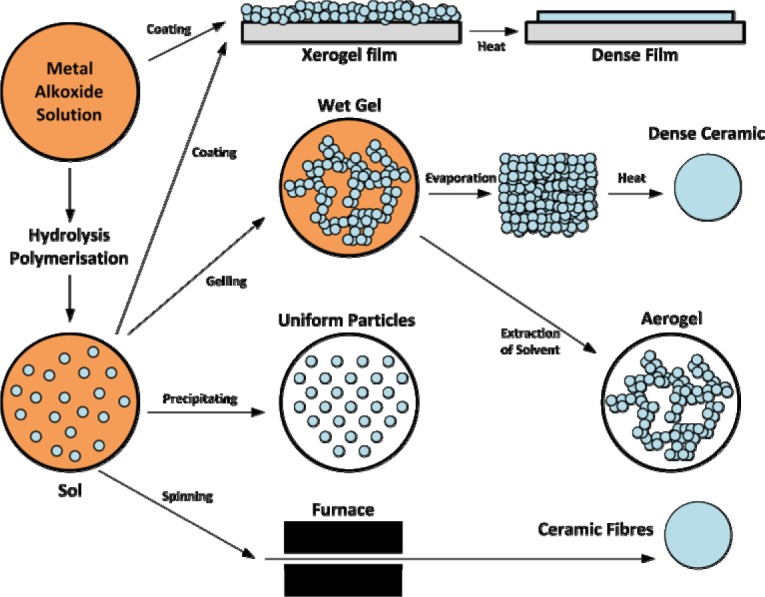
Description of technologies and products of the sol-gel process [68].

This process is carried out in water and organic solvents and its precursors are usually metal halides [64] (in organic solvents) and metal alkoxides such as Si(OR)_4_, SiR′(OR)_3_, Ti(OR)_4_, *etc*. [41,65–67] (Scheme 1). As seen in Scheme 2 [68], these materials are subject to a series of hydrolysis (or non-hydrolytic process in organic solvents) and condensation reactions that through nucleophilic substitution mechanisms result in sol formation. Sol is a colloidal solution in which individual particles interact weakly with each other and due to cross-linking reactions, converts into an integrated network (wet gel). This structure with further drying processes converts to gel.

In spite of silicon alkoxides, hydrolysis reactions of other metal alkoxides in water are too fast. The hydrolysis reaction of silicon alkoxides, is typically acid- or base-catalyzed [69]. As reported [70], silica networks formed under acid-catalyzed conditions are dense and those of formed in the presence of base are porous and loose.

**Scheme 3.**
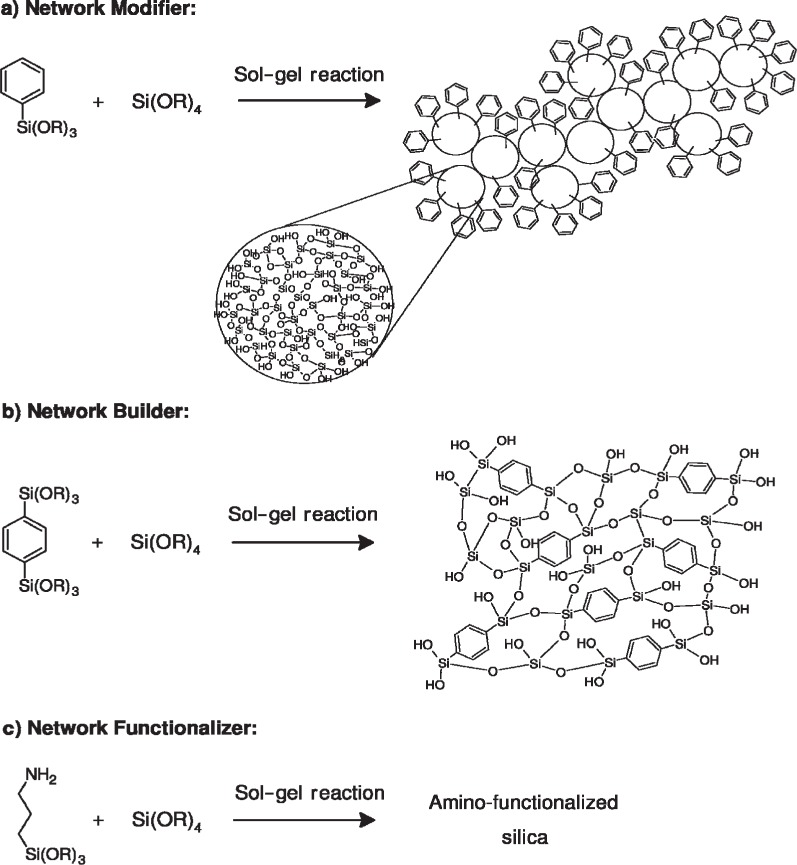
Organically functionalized trialkoxysilanes, R′Si(OR)_3_, used as (**a**) modifier; (**b**) builder and (**c**) functionalizer of silica-based network through the sol-gel process. Reproduced with permission from [72]. Copyright 2006 WILEY-VCH Verlag GmbH.

A suitable way for modifications of properties of materials obtained from this method is the introduction of organic groups (R) into their structure [71]. For example, the Si–C bond does not hydrolyze in most sol-gel processes and organic groups can be incorporated covalently into the network of inorganic gel using silsesquioxanes [(R–SiO_1.5_)*_n_* (*n* = even number)], such as R′(OR)_2_Si–R–Si(OR)_3_ compounds or derivatives of tetrafunctional silicon alkoxide [organically modified silicates (ORMOSILs)], such as TEOS, R′Si(OR)_3_, *etc*. [71]. If an organic group remains stable and does not hydrolyze during sol-gel processes (e.g., alkyl groups) it is defined as a network modifier. On the other hand, if it can react with another monomers or with itself (e.g., –CH=CH_2_) it is named as a network builder. Moreover, if it is a reactive functional group, such as –NH_2_, it is specified as a network functionalizer [72] (Scheme 3). These materials belong to class II interactions of organic-inorganic hybrid polymers.

*Another example of using sol*-gel processes is the synthesis of microporous zeolites (or molecular sieves). These materials are framework aluminosilicate and have a three-dimensional structure with high internal surface area and an ordered crystalline structure [73]. Zeolites with high silica contents are synthesized in the presence of bulky organic alkylammonium cations known as structure-directing agents or templates [74].

*Synthetic zeolites are micropo*rous (diameter of their pores < 2 nm) and have many applications such as membranes, catalysts, catalyst supports, gas storage, *etc*. [73]. In recent years, great efforts have been made on the direct preparation of hybrid organic-inorganic zeolites [75,76]. The incorporation of organic moieties within the pores or in the framework of microporous zeolites has developed their catalytic activity [77], molecular sieving property [78] and adsorption capacity [79].

### 2.2. Self-Assembly Process

**Figure 1.**
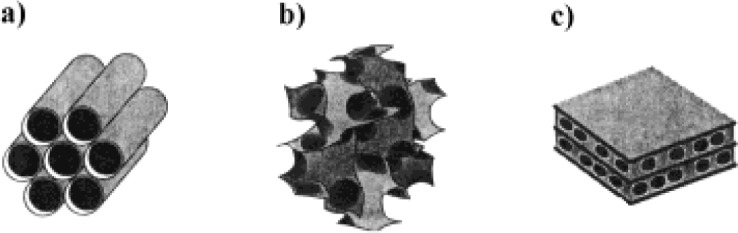
Representation of mesoporous M41S compounds including (**a**) MCM–41; (**b**) MCM–48 and (**c**) MCM–50. Reproduced with permission from [82]. Copyright 1999 WILEY-VCH Verlag GmbH.

In 1992, researchers of Mobil Oil Company discovered a new type of mesoporous silicas, so-called M41S [80,81]. M41S family includes hexagonal MCM–41, cubic MCM–48, and lamellar MCM–50 [82] (Figure 1). MCM is an abbreviation of “Mobil Crystalline Materials”.

Notwithstanding their amorphorous pore walls, these organic-inorganic hybrid compounds exhibit a long-range ordered array of uniform and controllable mesopores with sizes ranging between 2–10 nm [82]. Mesostructure compounds have high specific surface areas (greater than 1000 m^2^/g) [82] and the pore size of these materials enables them to adsorb much larger molecules than is possible for zeolites. These compounds are used for catalysis [83], gas adsorption [84], *etc*. M41S type compounds were synthesized using a silica source (e.g., TEOS) in the presence of long-chain alkyltrimethylammonium halide surfactants as templates or structure-directing agents [80]. These compounds formed through a base-catalysed hydrolytic sol-gel process. Mesostructures can be synthesized using different ionic or non-ionic surfactants or water-soluble polymers as templates [85,86]. Other types of ordered mesoporous silicas are Santa Barbara Amorphous (SBA) (e.g., SBA–15 [87]), Michigan State University (MSU) (e.g., MSU–2 [88]), folded sheets mechanism (FSM) (e.g., FSM–16 [89]), *etc*., respectively.

**Figure 2.**
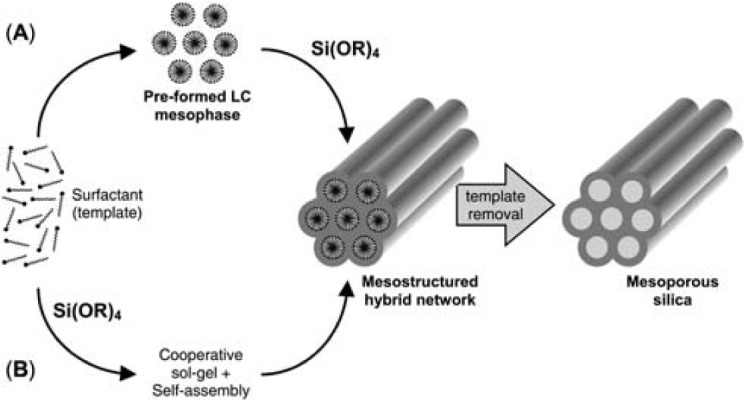
Synthesis of mesoporous compounds in the presence of template through (**A**) liquid-crystal template or (**B**) cooperative liquid-crystal mechanisms. Reprinted with permission from [91]. Copyright 2002 American Chemical Society.

In recent years, attempts of scientists resulted in syntheses of non-silica mesoporous metal oxides such as TiO_2_, ZrO_2_, Al_2_O_3_, Nb_2_O_5_, SnO_2_, mixed oxides such as SiAlO_3.5_, SiTiO_4_, carbon nanocage, mesoporous carbon nitride, *etc*. [90]. The formation of mesoporous materials is depicted schematically in Figure 2, based on “liquid crystal templating” mechanism [91]. Through route (a) at surfactant concentrations above its critical micelle concentration (CMC), the self-assembly of precursor molecules on the space between micellar rods of lyotropic liquid crystal phase and creates walls between them. Through route (b) at surfactant concentrations below CMC, the mesostructure is formed as a cooperative self-assembly of the precursor and surfactant molecules [82,91].

**Figure 3.**
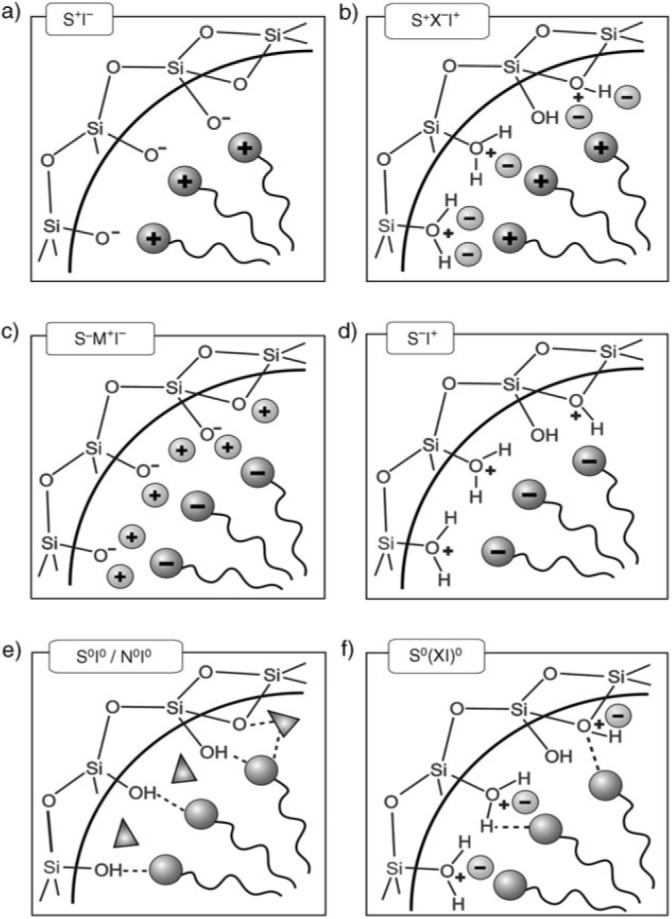
Different kinds of interactions between inorganic species and surfactant head groups through electrostatic interactions in (a) and (c) basic, (b) acidic and (d) neutral media or via hydrogen bonds between (e) unchared species or (f) ion pairs. Reproduced with permission from [93]. Copyright 2006 WILEY-VCH Verlag GmbH.

To prevent phase separation, there should be attractive interactions between inorganic precursors and template molecules. Depending on reaction conditions, such as pH and chemicals, e.g., inorganic precursors and templates, six routes have been suggested for syntheses of ordered mesoporous compounds [92]. These routes include (a) S^+^I^−^; (b) S^+^X^−^I^+^; (c) S^−^M^+^I^−^; (d) S^−^I^+^; (e) S^0^I^0^ or N^0^I^0^ and (f) S^0^(XI)^0^ where S is the surfactant, I is the inorganic phase and X^−^ is a mediator anionic species (usually a halide), M^+^ is a mediator cation, S^0^ is a neutral amine and N^0^ is a long-chain non-ionic template. Interactions between the charged inorganic phase and the head group of surfactants in routes (a–d) are mainly electrostatic forces; while those between neutral inorganic species and non-electrolyte template molecules are hydrogen bonds in routes (e) and (f) [92,93] (Figure 3). It is necessary to say that the inorganic precursor is neutral at its isoelectric point (e.g., at pH~2 for silica [94]) and has negative and positive electric charge at pH above and below this point, respectively.

In recent years, mesoporous silicas have been used as supports for preparations of organic-inorganic hybrid polymers and have many applications in lasers [95], drug delivery [96], pollutant adsorption [36], *etc*. In these compounds, different organic functional groups are located on the robust, thermally stable and porous inorganic substrate.

**Figure 4.**
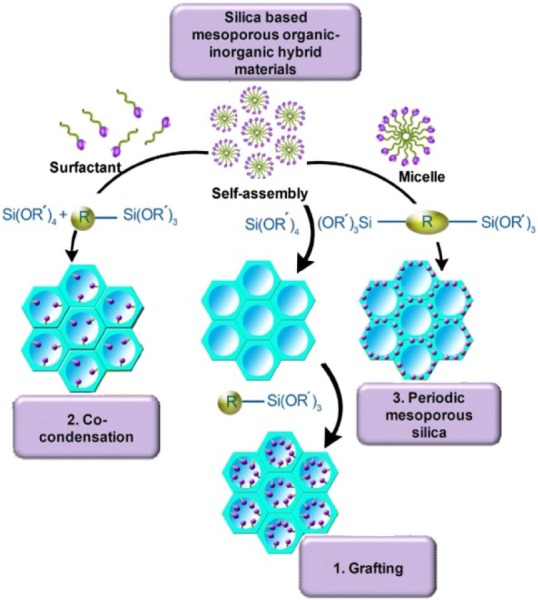
Different methods for the synthesis of organic-inorganic hybrid mesoporous silica: 1. Grafting, 2. Co-condensation or in situ grafting and 3. Periodic mesoporous silica. Reproduced wity permissiom from [93]. Copyright 2006 WILEY-VCH Verlag GmbH.

As illustrated in Figure 4, these organosilica compounds are prepared through (a) grafting or post-synthetic functionalisation; (b) co-condensation or one-pot synthesis and (c) synthesis of periodic mesoporous organosilicas (PMOs) [93,97,98]. In grafting processes, an organosilica is synthesized through condensation reaction between Si–OH groups of the pore walls of mesoporous silica and appropriate reagents such as, chlorosilanes (e.g., ClSiR_3_), alkoxyorganosilanes [e.g., RSi(OR′)_3_] *etc*. In the co-condensation method, an organosilica compound is prepared in one step through co-condensation reactions of tetraalkoxysilanes (e.g., TEOS) and an appropriate reagent (e.g., terminal trialkoxyorganosilanes) in the presence of a template. Syntheses of PMOs are carried out through the hydrolysis and condensation of bridged organosilane precursors of the type (R′O)_3_ Si–R–Si(OR′)_3_ in the presence of a structure-directing agent. In this method, R organic group are incorporated uniformly and periodically in the pore walls of resulted organosilica [93,98].

### 2.3. Assembling or Dispersion of Nanobuilding Blocks (NBBs)

The interest in nanoscale materials originates from this fact that their properties are a function of their size, composition, and structural order. Here, a series of compounds called as nanobuilding blocks (NBBs) are used for preparing organic-inorganic hybrid polymers. NBBs are capped with polymerizable ligands or connected through organic spacers, e.g., telechelic molecules or functional dendrimers [37]. These compounds maintain their integrity and fulfill certain (chemical or physical) functions in the final material. NBBs are classified as (1) clusters; (2) organically *in situ*- or post-functionalized nanoparticles; (3) layered compounds and (4) core-shell nanocompounds [37,99].

#### 2.3.1. Clusters

Metallic clusters (e.g., polyoxometalates) are an ensemble of bound atoms and contain a group of two or more metal atoms that bind together by direct Metal-Metal bonding. Clusters are connected either (1) by the polymerization of their ligands (e.g., allyl) and are called cross-linked clusters or (2) by coordinative bonds between coordinating groups of organic ligands (called as a linker or spacer) to each metal center (called as a connector) of clusters [100,101]. In the latter case, a linker is a bi-, tri- or tetradentate organic ligand (e.g., 1,4-benzenedicarboxylate) with an organic spacer between coordinating groups [102]. For example, a one-dimensional polymer is built when a metal cluster links to just one coordinating group of two bidentate organic linkers and through them links to other metal clusters. Coordination of more than two coordinating groups to each metal center, optionally through tri- or tetradentate linkers, results in two- or three-dimensional polymers [100]. The three-dimensional polymers are called metal-organic frameworks (MOFs) and their metal centers are bridged in three dimensions (Supporting information Figure S1). MOFs along with cross-linked clusters are major types of cluster-based organic-inorganic hybrid polymers [103,104].

#### 2.3.2. Organically *in Situ*- or Post-Functionalized Nanoparticles

In this method reactive organic groups are bound to the surface of inorganic moiety by strong ionic or covalent interactions. These organic groups are either grafted to preformed nanoparticles (called as post-functionalization) [105] or are incorporated during the synthesis of nanoparticles (called as *in-situ*-functionalization) [106]. This method is used for metal oxides (e.g., TiO_2_ [107]), chalcogenides (e.g., CdSe [108]), metallic nanoparticles (e.g., Au [109]) and polyoxometalates (e.g., γ-[SiW_10_O_36_]^8−^ [110]), *etc*.

#### 2.3.3. Layered Compounds

To improve the physical properties of polymers, inorganic compounds physically are dispersed in them (as fillers) in various forms such as platelets, spheres, fibers, *etc*. If the size of discrete inorganic components is in the range of 1–100 nm, the resulted organic-inorganic compound is called a nanocomposite [111]. Organic-inorganic hybrid nanocomposites are either as interpenetrating networks which will be explained in Section 2.4 [99] or layered inorganic components embedded into the organic matrix.

**Figure 5.**
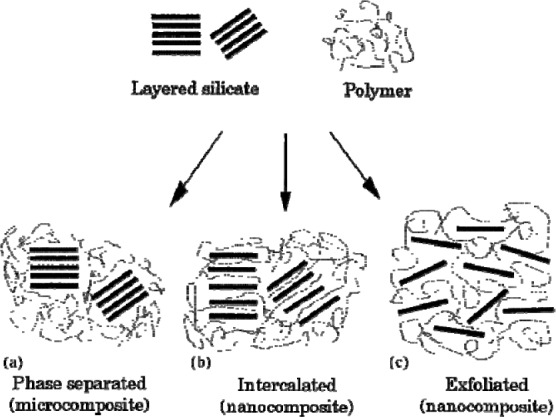
Schematic representation of different kinds of composites produced from interaction of layer compound with polymers: (**a**) phase separated; (**b**) intercalated and (**c**) exfoliated structures. Reprinted with permission from [116]. Copyright 2000 Elsevier.

Layered compounds are regular stacks of two-dimensional inorganic constituent. These compounds such as clay, are used as fillers in organic-inorganic hybrid composites. Some examples of nanocomposites are polyaniline-montmorillonite [112], polyaniline–V_2_O_5_ [113], polyvinyl alcohol-layered double hydroxide (LDH) [114], poly-N-vinyl carbazole-graphene oxide [115], *etc*. These compounds are incorporated in organic polymers as (a) a phase-separated structure in which the polymer can not intercalate within the inorganic layers and the inorganic component is dispersed as aggregates or particles within the polymer matrix; (b) an intercalated structure in which one or more polymer chains are inserted into the galleries (space between layers) of inorganic components and subsequently increase the interlayer spacing and (c) an exfoliated structure in which the insertion of polymer chains between inorganic sheets delaminates the layered compound [116] (Figure 5). Polymers can be intercalated to layered compounds by (1) solution; (2) melting polymers and (3) in situ polymerization of intercalated monomers [99]. However, some compounds (e.g., EDTA and malate) intercalate layer compounds [117,118] are purly nanocomposites and not organic-inorganic hybrid polymers.

#### 2.3.4. Core-Shell Nanocomposites

**Figure 6.**
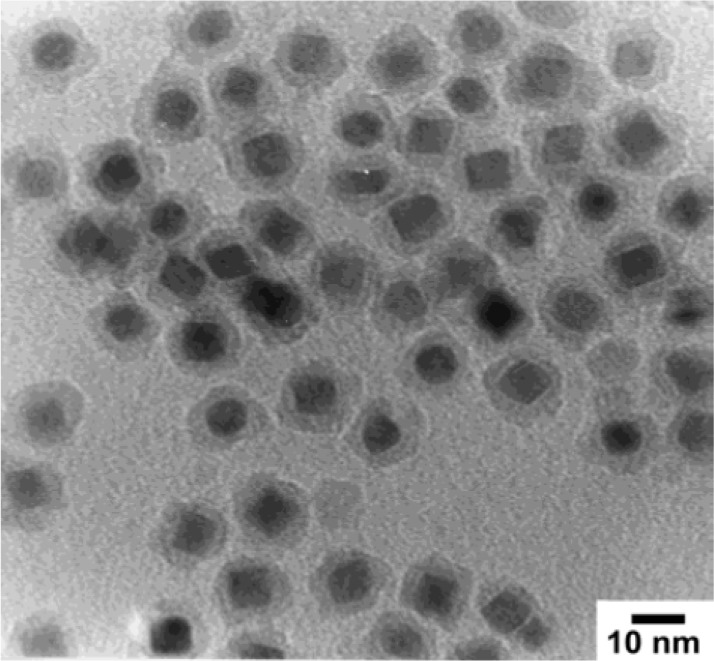
TEM of polystyrene-coated Fe_2_O_3_ particles. The polymer coatings are seen as a shell around the Fe_2_O_3_ cores. Reprinted with permission from [122]. Copyright 2003 American Chemical Society.

Core-shell nanocomposites (CSNs) consist of a core region covered by a shell domain (Figure 6). A series of CSNs are polymer-coated inorganic compounds [119–121]. The encapsulation of an inorganic particles in a polymer shell are carried out through (1) the adsorption of monomers on the surface of inorganic moiety followed by their polymerization in the adsorbed layer [122,123] and (2) interaction of inorganic particle with preformed polymer as in situ growing inorganic components in a polymer matrix [124]. CSNs are also shown as core@shell. Some kinds of CSNs are completely inorganic in nature (e.g., CdS@TiO_2_ [125]).

#### 2.3.5. Hierarchical Structures

**Figure 7.**
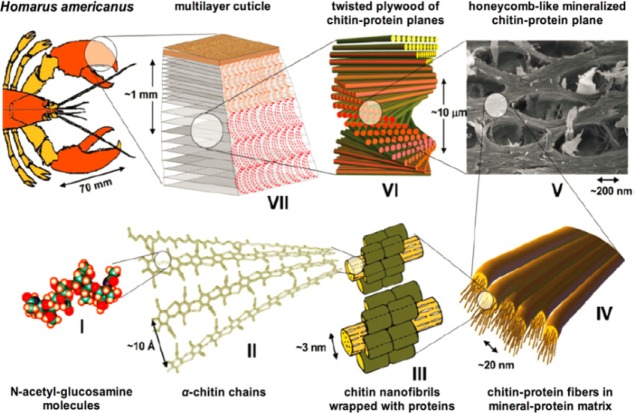
Hierarchical structure of lobster cuticle. Reproduced with permission from [127]. Copyright 2010 WILEY-VCH Verlag GmbH.

A hierarchy is a feature of self-assembly, where primary building blocks associate into more complex structures which are integrated into the next size level in the hierarchy. Self-assembly is emerging as an elegant, “bottom-up” method for the construction of nanostructured materials and is distinct from the template-directed assembly [126]. The feature of an integrated chemical, biological, *etc*. system is the assembly of components into a specific architecture that performs a certain function. Many examples of hierarchical structural designs are seen in nature and are characteristic of many self-assembling biological structures (e.g., lobster cuticle [127] and tendon [128]) (Figure 7).

Also, the hierarchy has resulted in the discovery of versatile nanoparticles with many applications [129], synthetic bone implant materials [130], mesostructured solid electrolytes [131], TiO_2_–graphene composite [132], *etc*. For example, the incorporation of interconnected macropores in mesoporous films of TiO_2_–graphene composite (Supporting information Figure S2) augments the capacity of composite film for adsorption and photodegrading organic molecules and can be used for the treatment of waters.

### 2.4. Interpenetrating Networks (IPNs)

**Figure 8.**
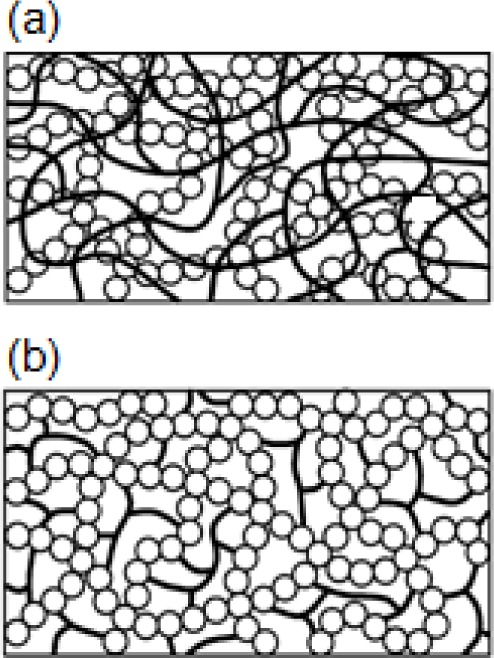
Schematic representation of IPNs (**a**) without chemical bonds between moieties and (**b**) dual hybrid network. Reprinted with permission from [99]. Copyright 2003 Elsevier.

IPNs comprise organic and inorganic networks that are microscopically phase-separated. However, they seem uniform macroscopically and there are weak interactions between moieties of IPNs (class I interactions). These components are partially interlaced and, upon separating IPNs, chemical bonds are broken. IPNs are synthesized by (1) the formation of a secondary network in a primary one (a sequential two-step process); (2) the simultaneous formation of two networks and (3) IPN components connect to each other through covalent bonds in which the compound is named a dual organic-inorganic hybrid polymer (class II interactions) [99] (Figure 8). In the first method, IPNs are prepared by polymerization in a sol-gel network [133] or sol-gel process in the presence of preformed polymer [134]. In the third case, either bifunctional precursors are used or a preformed inorganic or organic polymer are functionalized by the required functional groups for the formation of other types of networks [135].

But, when three-dimensional frameworks such as zeolites or mesoporous silicates, are used as inorganic components and polymer is formed without crosslinking, the polymer chains could be partially or completely removed from the product. In this case, the composite is called pseudo-interpenetrating polymer network (PIPN) [136,137].

At the end of this section, it is noteworthy that the inclusion of metals in organic-inorganic hybrid polymers occurs at the molecular scale and the incorporation of metal complexes and metallic ions in polymers by coordination interactions does not produce organic-inorganic hybrid polymers [99]. For example, metal-centered polymers [138] and products of coordination of metallic ions to the polymer backbone [139] and polymerization of metal-coordinated monomers [140] are called metal-containing polymers [99].

## 3. Removal of Heavy Metals by Organic-Inorganic Hybrid Polymers

Based on the classifications of these kinds of compounds in the previous section, by analysis some comprehensive references, we study their synthesis methods, structure and mechanisms of heavy metal adsorption from water or wastewater.

### 3.1. Adsorption of Heavy Metals Using Materials Synthesized from Sol-Gel Method

As extensive researches have been done on producing silica-based compounds by the sol-gel method, these kinds of materials are discussed here. These compounds can be synthesized either directly through polymerization reaction of their precursors [141] or grafting appropriate functional groups on parent materials formed from sol-gel method such as silica gel and some examples [142–167] are given in Table 1.

**Scheme 4.**
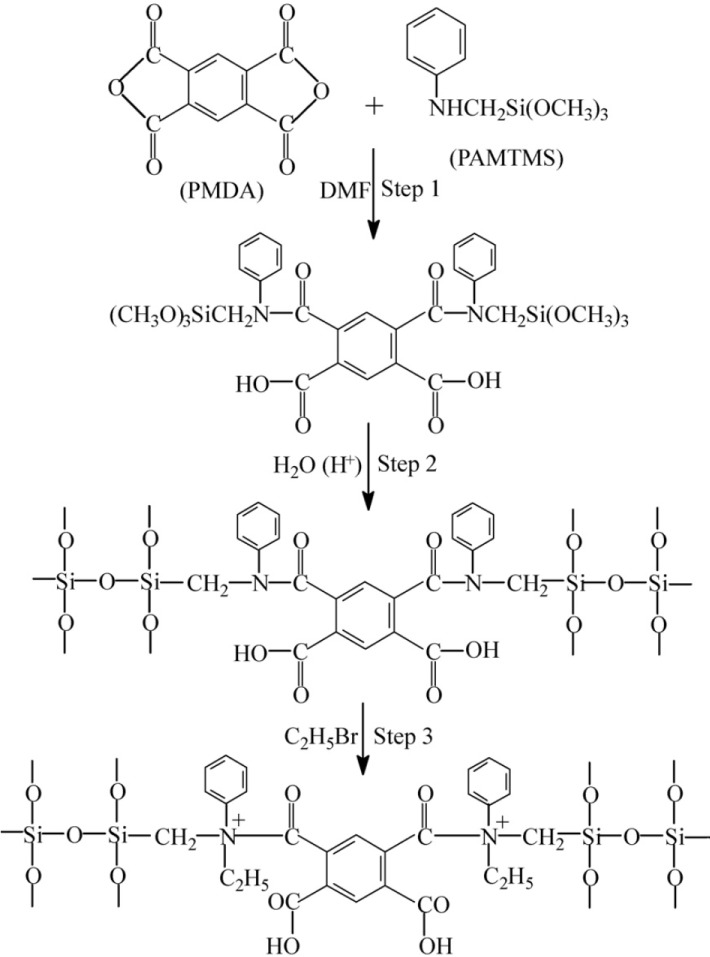
The preparation steps of zwitterionic hybrid polymers. Reprinted with permission from [142]. Copyright 2010 Elsevier.

Zwitterionic hybrid polymers [142,143] were synthesized on the basis of ring-opening polymerization of pyromellitic acid dianhydride (PMDA) (a network builder) and phenylaminomethyl trimethoxysilane (PAMTMS), followed by the amination (Scheme 4).

Ionic groups of opposite charges on the polymer chain (quaternary amines and carboxylic groups) allowed their electrostatic interactions with heavy metals. TGA analysis showed a higher PDMA content of prepared samples, increased their thermal degradation temperature [143].

Kinetics experiments showed about 80% of Pb^2+^ and Cu^2+^ ions were adsorbed after approximately 3 h. Kinetics and thermodynamics measurements of Cu^2+^ and Pb^2+^ were carried out at pH = 4 and 5, respectively in which these metal ions exist mainly as their free forms.

Some researchers prepared imprinted ionic polymers (IIPs) for adsorption of metal ions [144–147]. IIPs were prepared by either the functionalization of a support surface (e.g., silica gel) using an organic chelating group and subsequently imprinted with metal ions as a template or by the co-condensation of monomers containing functional groups (in the presence of metal ions) and then co-condensation of them by sol-gel processes and finally releasing metal ion from the prepared compound. For example, thiocyanato- [144] and amino-functionalized [145] monomers were used to synthesize Cd^2+^-imprinted polymers. These compounds showed high adsorption capacity and high selectivity toward templating ions.

SEM images of Cd^2+^-imprinted and non-imprinted amino-functionalized hybrid polymers show that the former exhibits the porous feature [145]. The amino-functionalized Cd^2+^-imprinted adsorbent [145] had rough surface and smaller size and thus larger surface area (152 m^2^/g), compared to its non-imprinted form (101 m^2^/g). Optimum pHs for the adsorption of Cd^2+^ were in the range of 4–8 where adsorption sites were deporotonated and Cd^2+^ ions were not hydroxilated. Kinetics experiments showed that the saturation of these sites was after about 20 min. The *q*_max_ (maximum adsorption capacity) value for the adsorption of Cd^2+^ on adsorbent was 77.2 mg/g. An IIP synthesized using mercapto-functionalized monomers [146] was applied for the separation of Cd^2+^ from Ni^2+^, Cu^2+^ and Zn^2+^ that have similar ionic radii. The *relative selective factor values* of Cd^2+^/Ni^2+^, Cd^2+^/Cu^2+^ and Cd^2+^/Zn^2+^ (imprinted relative to non-imprinted) were 41.687, 65.617 and 66.937, respectively. The *q*_max_ values for the adsorption of Cd^2+^ on Cd^2+^-imprinted and non-imprinted adsorbents were 83.89 and 35.91mg/g, respectively which were due to larger surface area of Cd^2+^-imprinted adsorbent and its selectivity toward Cd^2+^.

For a Fe^3+^-imprinted adsorbent [147] prepared by grafting silica gel with cyanato functional groups, the *q*_max_, optimum pH, and the time for the equilibrium binding of Fe^3+^ on the Fe^3+^-imprinted adsorbent were 35.6 mg/g, 3 and 20 min, respectively. The selective coefficient values of Fe^3+^/Co^2+^, Fe^3+^/Pb^2+^, Fe^3+^/Cd^2+^ and Fe^3+^/Ni^2+^ of the Fe^3+^-imprinted adsorbent were 11.95, 50.71, 16.96 and 8.56 times greater than those of a non-imprinted adsorbent, respectively. Due to the high affinity of these functional groups for Fe^3+^ and Cd^2+^, the adsorption rate in these kinds of adsorbents is rapid. As reported by a number of authors [144−147], they washed the used imprinted adsorbents with 3 M HCl and reused the regenerated adsorbent repeatedly without significant loss of adsorption capacity.

**Scheme 5.**
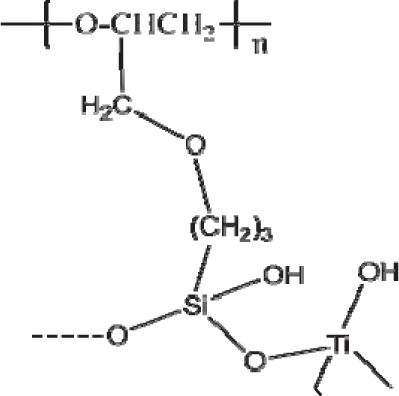
Silicon and titanium-contained adsorbent. Reprinted with permission from [148]. Copyright 2013 Taylor and Francis.

Some researchers are interested in synthesizing silicon and metal containing compounds [148,149] which one of them is poly-GPTS/Ti(O)OH [148]. Poly-GPTS is prepared from the hydrolysis of 3-glycidyloxypropyltrimethoxysilane (GPTS) and poly-GPTS/Ti(O)OH from the hydrolysis of poly-GPTS and titanium isopropoxide, respecyively (Scheme 5).

According to the kinetics experiments, the adsorption equilibrium was attained after about 10 min. Their experiments showed that at pH ≈ 5.5, Pb^2+^, Cu^2+^ and Cd^2+^ interact with oxygen atoms of –OH groups of these adsorbents. Authors believed that due to stronger interactions between metal ions and –OH groups of poly-GPTS/Ti(O)OH, the *q*_max_ values for the adsorption of Pb^2+^, Cu^2+^ and Cd^2+^ on poly-GPTS/Ti(O)OH (199, 42.79 and 39.41 mg/g, respectively) were bigger than those of poly-GPTS.

**Figure 9.**
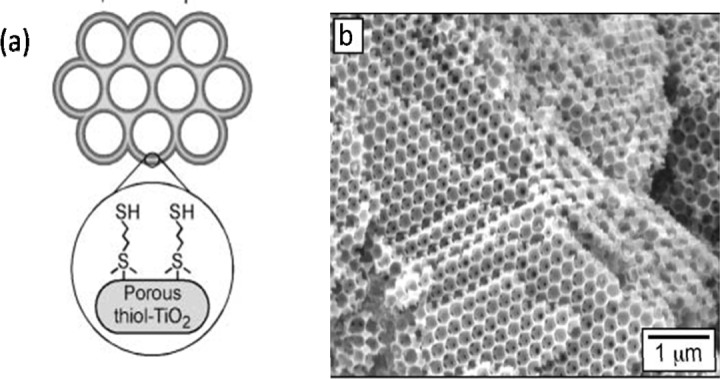
(**a**) Scheme of macroporous thiol-titania material with propyl-siloxane linkages and (**b**) its SEM image. Reproduced with permission from [149]. Copyright 2002 The Royal Society of Chemistry.

Another example of metal containing compounds is a series of macroporous thiol-functionalized titania and zirconia frameworks with propyl-siloxane, ethyl-sulfonate and propyl-sulfonate linkages [149] (Figure 9). The *q*_max_ values for the adsorption of Pb^2+^ and Hg^2+^ were in the ranges of 0.27–0.82 and 0.33–1.41 mmol/g, respectively.

The ratio of adsorbed moles of Hg^2+^ and Pb^2+^ per mole of –SH groups (or their adsorption efficiency) were in the ranges of 0.19–0.82 and 0.21–0.72, respectively. This showed that the inner surface of these compounds were not completely accessible for these metal ions and had a smaller *q*_max_ value for Pb^2+^ compared to that of Poly-GPTS.

Xerogels [150,151] and aerogels [152] have been used for the adsorption of heavy metals [150,151]. Lima *et al*. [150] used mesoporous 7-amine-4-azahepthylsilica and 10-amine-4-azadecylsilica xerogels (abbreviated as AAH–Si and AAD–Si, respectively) for the adsorption of Pb^2+^ on which silica gels were functionalized with –(CH_2_)_3_–NH–(CH_2_)_3_–NH_2_ and –(CH_2_)_3_–NH–(CH_2_)_6_–NH_2_ groups, respectively. AAH–Si and AAD–Si interacted with Pb^2+^ through their amine groups and *q*_max_ values for the adsorption of Pb^2+^ on AAH–Si and AAD–Si were 36.64 and 30.27 mg/g, respectively and the equilibrium time of process was about 120 min. The higher adsorption capacity of AAH–Si over AAD–Si was due to the ability of AAH–Si to form a stable six-member ring with Pb^2+^ whereas AAD–Si was expected to form a nine-member ring that shows a transannular strain. In another work, an aniline-functionalized silica xerogel [151] was used for adsorption of Ni^2+^ and Mn^2+^ at pH 4–5. The adsorption equilibrium was achieved after about 20 and 30 min for Ni^2+^ and Mn^2+^, respectively. The enthalpies of adsorption, obtained from calorimetric measurements, showed that weak interactions occur between xerogel and metal ions. It implied that the steric hindrance of phenyl group limits the accessibility of amine groups towards metal ions. Similar to above-mentioned xerogels, an amino propyl-modified aerogel used for adsorption of Pb^2+^ and Cd^2+^ [152]. However, in spite of xerogels, the equilibrium time was 24 h.

Some efforts were done for the separation of metal ions from ethanol [153,154]. Qu *et al*. [153] investigated it through functionalization of silica gel using a series of amino-terminated dendrimer-like polyamidoamine (PAMAM) polymers. This process was endothermic and the Cu^2+^ adsorption capacities of these compounds with different amino contents were in the range of 9.8–78.7 mg/g. Kinetic experiments showed its adsorption equilibrium time was about 10 h. Pissetti *et al*. [154] synthesized an ethylenediamine-modified poly(dimethylsiloxane) elastomeric network (Pen) for the adsorption of Cu^2+^, Fe^3+^ and Ni^2+^ from ethanol. In these compounds, the ethylenediamine functional groups were located at the nodes of the networks constituted by silsesquioxane clusters. The nodes of the polymeric network were constituted of clusters rich in CSi(OSi)_3_ units. The interacting groups of this polymer were amine groups too (Supporting information Figure S3). Also, characteristics of a number of adsorbents [155–167] prepared by sol-gel method, have been given in Table 1.

### 3.2. Adsorption of Heavy Metals on Organically-Functionalized Mesoporous Compounds

In this section, adsorption capacities and operation conditions of silica-based mesopores are studied. As referred in Section 2.2, these compounds are synthesized through grafting, co-condensation or in situ grafting of mesopores and by synthesis of periodic mesoporous silicas. Details of a number of examples [168–198] are given in Table 2. One characteristic of mesoporous compounds is their high surface area. For example, it was shown that [168] when under similar conditions SBA–16 mesoporous compound was used or preparation of organic-inorganic hybrid compounds, its *q*_max_ values for adsorption of Cu^2+^ and Co^2+^ were higher than those of adsorbent prepared by silica gel.

**Figure 10.**
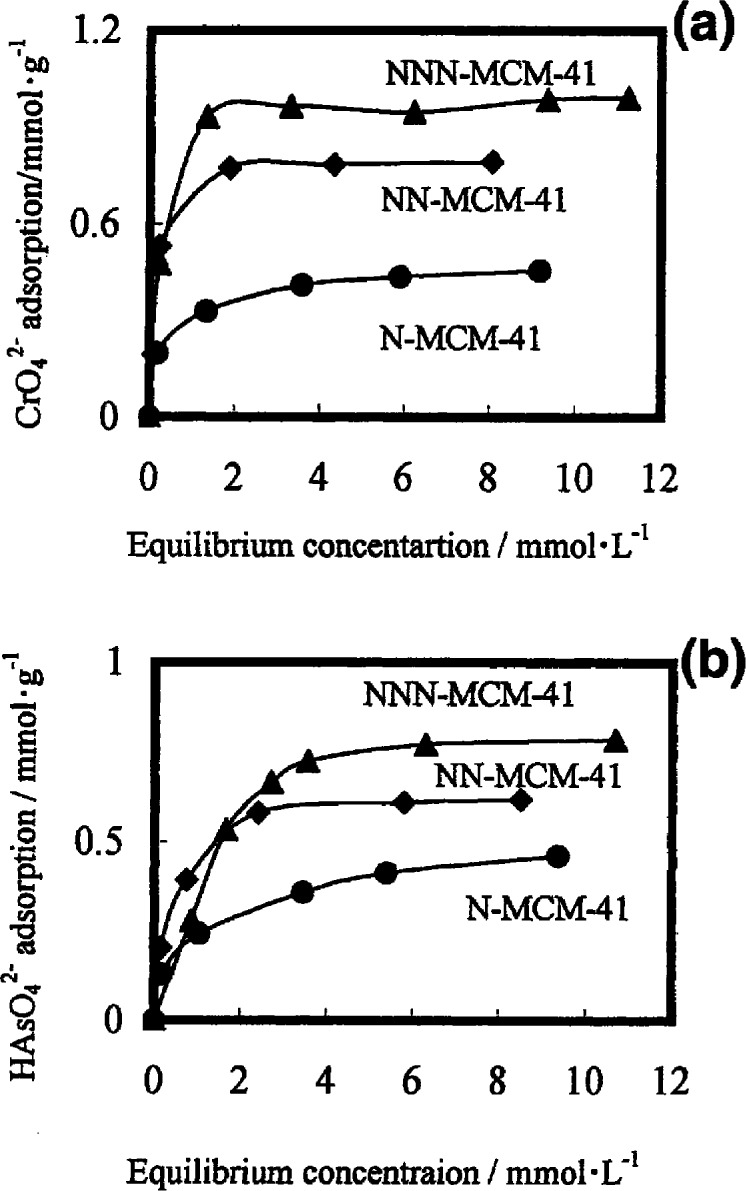
Adsorption isotherms of (**a**) arsenate and (**b**) chromate on mono-, di- and triamino-functionalized MCM–41 which are shown with N–MCM–41, NN–MCM–41 and NNN–MCM–41, respectively. Reprinted with permission from [169]. Copyright 2002 American Chemical Society.

Another factor affecting the adsorption capacity of an adsorbent is the density of its functional groups [169,170]. Yoshitale *et al*. [169] studied and compared effects of functional group density on the adsorption of chromate and arsenate oxyanions and other heavy metals. The H_2_N–(CH_2_)_3_–, H_2_N–(CH_2_)_2_–NH–(CH_2_)_3_– and H_2_N–(CH_2_)_2_–NH–(CH_2_)_2_–NH–(CH_2_)_3_– groups were grafted on the surface of MCM–41and SBA–1 to prepare their mono-, di- and triamino-functionalized derivatives, respectively [169]. These amino groups are abbreviated as N–, NN– and NNN–, respectively. They showed that the pore size and the surface area of these compounds decrease with the increase of their amine density and similar results have been reported for mono-, di- and triamino-functionalized SBA–15 [170]. Experimental results implied that the interaction between the amine groups and oxyanions was ionic and all amine groups can involve in the adsorption process. Thus, *q*_max_ values for the adsorption of CrO_4_^−^ and HAsO_4_^2−^ increase from mono- to triamino-functionalized derivatives of both MCM–41 and SBA–1 (Figure 10) [169]. As reported [169,171,172], amino groups under acidic conditions had much higher *q*_max_ for the adsorption of arsenate. At the adsorption saturation, the stoichiometries of oxyanions to N atoms were 0.5 for all adsorbents derived from SBA–1, whereas they were 0.25–0.35 for MCM–41 derivatives [169]. This was due to, on SBA–1, all amino heads were randomly distributed and acted as adsorption sites but, on MCM–41, they formed domains on MCM–41.

Arencibia *et al*. [170] compared effect of synthesis condition on the adsorption of a number of metallic ions by monoamino-functionalized SBA–15 prepared by calcination, extraction and further hydration of calcined silica. They showed that *q*_max_ values of these adsorbents for Ni^2+^, Cu^2+^, Zn^2+^, Cd^2+^ and Pb^2+^ were different from each other and there was no relation between the pore size and surface area of mesopores with their *q*_max_ values for the adsorption of metallic ions from solutions. Also, different functional groups on SBA–15 can adsorb different metal cations [173–177].

**Figure 11.**
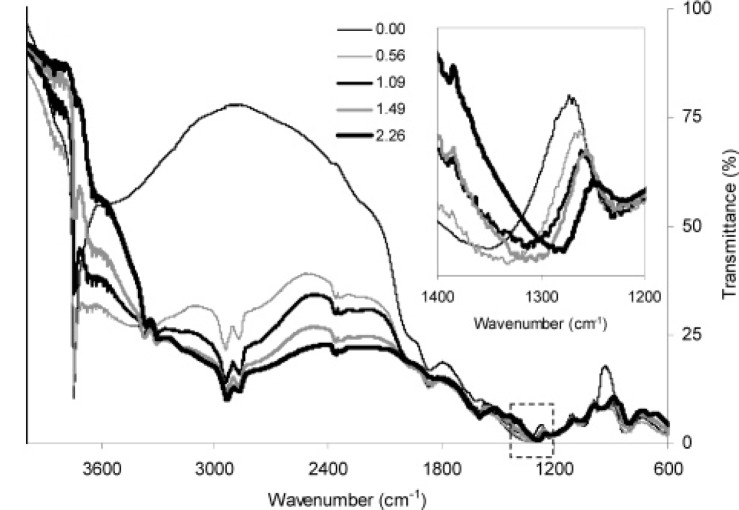
FTIR spectrum of NH_2_–MCM–41 with different NH_2_ loadings. The insert figure displays the peak shift caused by the interaction between the amino and surface silanol groups. Reprinted with permission from [178]. Copyright 2006 American Chemical Society.

In some works, amino-functionalized MCM–41 mesopores were used for adsorption of heavy metal cations [178–180]. Yeoung *et al*. [178] studied the steric hindrance effect on the adsorption of Au^3+^, Cu^2+^ and Ni^2+^ on NH_2_CH_2_CH_2_CH_2_–, CH_3_CH_2_CH_2_NHCH_2_CH_2_CH_2_– and (CH_3_CH_2_CH_2_)_2_NCH_2_CH_2_CH_2_–functionalized MCM–41 which their abbreviated names are NH_2_–MCM–41, NHR–MCM–41 and NR_2_–MCM–41, respectively. The results showed that only Au^3+^ (from the mixture of these ions) was selectively adsorbed on these compounds, resulted from interactions of Au^3+^ ions with lone pair electrons of N atom of amine groups. They observed that the adsorption capacity of Au^3+^ decreased from NH_2_–MCM–41 to NR_2_–MCM–41 which was due to the increase in steric hindrance of these spacious groups on the lone-pair electrons of N atoms. Authors showed that Au^3+^ was adsorbed on NH_2_–MCM–41 with the amine loading ratios higher than 0.6 mmol/g. This was because, at low amine loading ratios (<0.6), strong H-bonding between silanol and amine groups prevented interactions of amine groups with Au^3+^ ions whereas at the amine loading ratio higher than 0.6 amine groups formed patches of amino-derived areas and this destroys H-bonding between silanol and amine groups which was revealed at the peak shifting of around 1300 cm^−1^ in the FT-IR spectra (Figure 11). The used NH_2_–MCM–41 was washed by 5 M HCl and all the adsorbed gold ions were recovered and the regenerated adsorbent retained its adsorption capacity after repeating usages.

In another work, researchers changed contents of functional groups and used different ratios of 3-mercaptopropyl triethoxysilane (TMMPS) and TEOS to synthesize thiol-functionalized SBA–16 (SH–SBA–16) samples [181]. With the increase in the sulfur content of samples, –SH groups on the surface partially blocked the adsorption of nitrogen molecules and decrease their surface areas and pore sizes and the adsorption capacity of SH–SBA–16 samples increases. The used SH–SBA–16 sample for the adsorption of Cu^2+^ was recovered by 1 M HCl and after the seventh stripping cycle, its adsorption capacity was 90.5% of its initial value.

The adsorption of Cu^2+^ on aminopropyl-substituted SBA–15 (SBA–15–N) and a temperature-aged sample (SBA–15–N–T) was studied by Soler–Illia *et al*. [182]. It was observed that upon the addition of functional groups, the surface area of SBA–15–N and SBA–15–N–T decrease compared to SBA–15. Experiments showed that the *q*_max_ value for the adsorption of Cu^2+^ on SBA–15–N was bigger than that of SBA–15–N–T which was due to the rearrangement of inorganic network in SBA–15–N–T. Due to the thermal treatment of SBA–15–N intensive H-bond interactions between its amino groups and the surface silanols disrupted and this improved the accessibility of amino groups on the pore surface.

**Figure 12.**
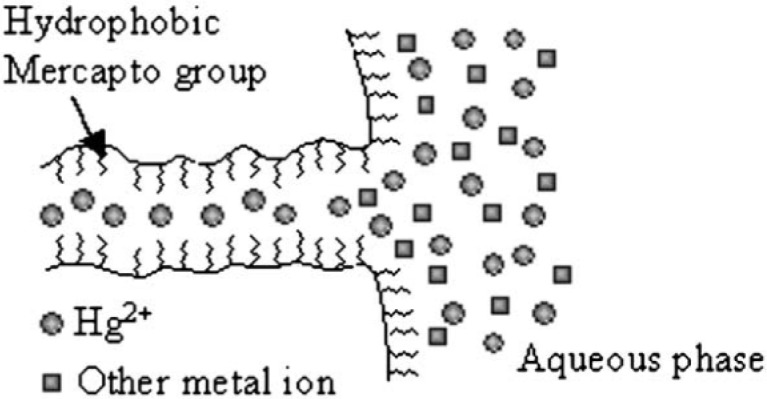
Difference between adsorption mechanism of Hg^2+^ and other metallic ions. Reprinted with permission from [184]. Copyright 2001 Elsevier.

Dai *et al*. [183] studied effect of hydrophobicity of functional groups on their adsorption capacity. HMS, SBA–15 and PME (PMO material bridged with ethylene groups) functionalized with *N*-((trimethoxysilyl)propyl)-*N*,*N*,*N*-tri-*n*-butylammonium (TSPBC) and *N*-((trimethoxysilyl)propyl)-*N*,*N*,*N*-trimethylammonium (TSPMC) ions were used for the adsorption of hydrophobic ReO_4_^−^ ions [183]. The results showed that TSPBC-functionalized HMS, SBA–15 and PME had bigger *q*_max_ values for the adsorption of ReO_4_^−^ than those functionalized with TSPMC. This was due to the higher hydrophobic character of butyl groups compared to methyl groups that enhanced the adsorption of ReO_4_^−^ ions. When a cationic surfactant (here CTAC) was used for synthesizing mesopores (called as MEB sample), its chloride ions attached to the protonated silanol groups and anion-exchange sites and hindered the adsorption of ReO_4_^−^ ions. To avoid high chloride content in these mesopores, authors used TSPBC and a neutral surfactant (rather than CTAC) to synthesize mesoporous compound (called as NMEB) that increased greatly its *q*_max_ for the adsorption of ReO_4_^−^ ions compared to MEB. In another work, a bifunctional HMS, prepared by mercaptopropyl and aminopropyl groups, was used for the adsorption of Hg^2+^, Cu^2+^ and Cd^2+^ [184]. Heavy Metal ions were adsorbed by –SH heads of mercaptopropyl groups. Due to higher affinity between –SH group and different mechanisms of its adsorption, the Hg^2+^ adsorption and its adsorption selectivity increased greatly compared to Cu^2+^ and Cd^2+^ (Figure 12). Hg^2+^ can be adsorbed on the inner surface of pores whereas Cu^2+^ and Cd^2+^ were adsorbed on the outer surface of adsorbent or near the pore openings. As reported [36,180,185–187], −SH groups of organically-functionalized mesopores have a great *q*_max_ for adsorption of Hg^2+^. An example of a compound with two functional groups is 1-benzoyl-3-propylthiourea-functionalized MCM–41 (MCM–41 BTU) which was synthesized through a two-step modification process [188] to better control removal of Hg^2+^ and to prevent unnecessary pore blocking. In the first step they prepared 3-aminopropyl-MCM–41 (MCM–41 NH_2_) and in the second step, they prepared MCM–41 BTU using MCM–41 NH_2_. Their surface area and pore size were decreased as in order as MCM–41 > MCM–41 NH_2_ > MCM–41 BTU.

**Figure 13.**
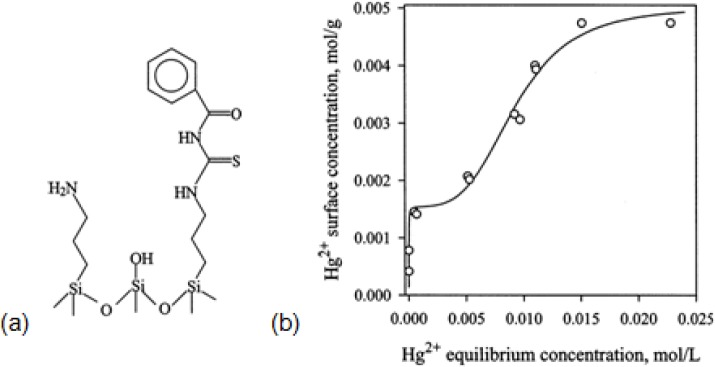
(**a**) Functional groups of MCM–41BTU and (**b**) adsorption isotherm for adsorption of Hg^2+^ on MCM–41 BTU. The initial sharp part of adsorption isotherm is related to adsorption by 3-aminopropyl residuals. Reprinted with permission from [188]. Copyright 2003 Springer Science and Business Media.

The concentration of 1-benzoyl-3-propylthiourea and (unreacted) aminopropyl groups on the surface of MCM–41 BTU were 1.5 and 0.65 mmol/g, respectively (Figure 13). The adsorption isotherm of Hg^2+^ on MCM–41 BTU had two regions (Figure 13). In the first region, Hg^2+^ strongly were adsorbed on 3-aminopropyl residuals (*K*_1_ = 1.41 × 10^5^ M^−1^ and *q*_max_ = 1.55 mmol/g) and then in the second region the ions were adsorbed on the 1-benzoyl-3-propylthiourea residuals (*K*_2_ = 1.08 × 10^2^ M^−1^ and *q*_max_ = 3.48 mmol/g).

Zhao *et al*. [189] synthesized a number of two-dimensional disulfide-bridged periodical mesoporous organosilicas (PMO) with high sulfide contents for the adsorption of Hg^2+^. They used bis(triethoxysilylpropyl) disulfide (BTSPDS) and TEOS as precursors Zn(NO_3_)_2_ in a number of syntheses. XRD patterns demonstrate that in the presence of Zn^2+^ highly ordered hexagonal disulfide-bridged PMO composites were obtained. Eventually, Zn^2+^ ions were etched off in a strong acid. XRD patterns of the Zn^2+^ treated compounds showed their high hydrothermal stability which was enhanced with the increase in their BTSPDS/TEOS ratios. Hg^2+^ ions interacted with sulfur atoms of –S–S– bridges of adsorbents. Adsorption capacities of these compounds for Hg^2+^, similar to those of tetrasulfide-functionalized polyvinylpyrrolidone (PVP)/SiO_2_ composite mesopores [190] or disulfide-functionalized SBA–1 [191], were high and increased in their BTSPDS content [189]. This shows that double sulfide functioalized mesopores are emerging candidates for adsorption of Hg^2+^.

Also, other kinds of mesoporous compounds with various functional groups were used for adsorption of heavy metals [192–198]. Yeung *et al*. [195] increased selectivity of an aminopropyl-grafted MCM–41 (NH_2_–MCM–41) for the adsorption of Ni^2+^ and Cd^2+^ through chelation of these ions. The isoelectric point of NH_2_–MCM–41 was 3.2. Below this pH, the adsorbent did not adsorb Ni^2+^ and Cd^2+^ and at pH = 5, its *q*_max_ values of Cd^2+^ and Ni^2+^ were 0.71 and 0.69 mmol/g, respectively. In binary mixtures of Ni^2+^ and Cd^2+^ at pH > 3.2, the *q*_max_ values of NH_2_–MCM–41 for Ni^2+^ and Cd^2+^ were 0.60 and 0.16 mmol/g, respectively. The authors tuned the selectivity of adsorbent by an appropriate chelate, EDTA. EDTA selectively bound to Ni^2+^ (as NiEDTA^2−^ complex) and changed its adsorption selectivity toward Cd^2+^. Authors tried to separate these ions by adding EDTA and adjusting the pH of solution. At pH = 2, only Ni^2+^ ions (as NiEDTA^2−^) were adsorbed by –NH_3_^+^ headgroups in mesopores and, at pH = 5, only Cd^2+^ ions were adsorbed by –NH_2_ groups of adsorbent (Supporting information Figure S4).

The H_2_N(CH_2_)_2_NH(CH_2_)_3_–functionalized MCM–41 and MCM–48 (abbreviated as NN–MCM–41 and NN–MCM–48) [196] were used for the adsorption of Fe^3+^, Cu^2+^, Co^2+^ and Ni^2+^. Then, the authors applied the used mesopores for the adsorption of arsenate. The Fe^3+^, Cu^2+^ and Co^2+^ ions bound to ethylenediamine (en) headgroups of mesoperes as Fe(en)_2_, Cu(en)_2_ and Co(en) whereas Ni^2+^ interacted with surface silanol groups. The adsorption capacities of NN–MCM–48 for these metal ions were much larger than those of NN–MCM–41. Also, it was shown that Cl^−^ and SO_4_^2−^ anions inhibited arsenate adsorption on these kinds of adsorbents by different extents.

### 3.3. Adsorption of Heavy Metals on Composites of Layered Compounds with Organic Polymers

As pointed in Section 2.3.3, these kinds of compounds are prepared as phase separated, intercalated and exfoliated structures [118]. Here, we study a number of works carried out regarding their abilities for the adsorption of heavy metals. Some examples [199–232] are summarized in Table 3.

**Figure 14.**
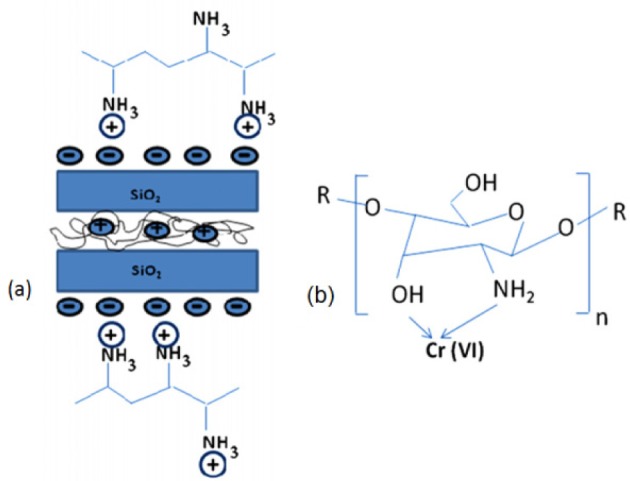
(**a**) Schematic structure of chitosan and clay hybrid and (**b**) mechanism of interaction of closite 10 A/chitosan nanocomposite (CCN) with Cr(VI). Reprinted with permission from [199]. Copyright 2011 Elsevier.

Chitosan is a natural polycationic biopolymer that can chelate with heavy metals by its amino and hydroxyl groups and its nanocomposites with clays and zeolites are used for adsorption of heavy metals. However, it has a low surface area, with weak chemical and mechanical properties. Thus, physical and chemical modifications are necessary to overcome these limitations. On the other hand, the clay has a lamellar structure with negatively charged surface that interacts with polycationic chitosan. There is high possibility that one or both surfaces of the clay layers can be modified by chitosan. Chitosan-functionalized closite 10A [199], bentonite [200], perlite [201–204], clinoptilolite [205], alumina [206,207], montmerilonite [208,209] and calciul alginate [210] were used for the adsorption of heavy metals from water. In these compounds, functional groups of chitosan (–NH_2_ and –OH) interact with heavy metals. In spite of cellulose/hydyoxyapatite in which negative sites of hydroxylapatite are interacting sites of nanocomposite [211]. XRD showed the interlayer spacing of chitosan-functionalized bilayer compounds increases, which showed a complete [199] or partial exfoliated [209] surface morphology. Electrostatic interaction between chitosan and negatively-charged surface of layer compounds such as closite A [199], perlite [203,204] and, alumina [206,207] resulted in the formation of nanocomposites. As an example for the adsorption of heavy metals by these kinds of compounds, authors studied the adsorption of Cr(VI) polyanions by closite 10 A/chitosan nanocomposite (CCN) (Figure 14) [199]. The TEM image demonstrated the formation of exfoliated surface morphology. The zeta potential of CCN surface at pH below eight was positive because the amine groups of chitosan were protonated at pH below eight and Cr(VI) ions existed as Cr_2_O_7_^2−^, CrO_4_^2−^ and HCrO_4_^−^ polyanions at pH range of 2–6. Thus, The optimum pH for the adsorption process was found to be 3. Similarly, nanocomposites of chitosan with perlite [201], alumina [206,207], montmorillonite [208], with Cr(VI) oxyanion or chitosan-clay with selenate [209] occurs via their protonated amino groups. As reported [200,202–205,210] chitosan-layered nanocomposites interaction with metal cations occurs through –OH and –NH_2_ groups of chitosan. The *q*_max_ values of these adsorbents completely depend on the used layered compounds, Table 3. The used CCN was regenerated by washing with 0.01 N H_2_SO_4_ and, after four cycles, its adsorption capacity was 78.47% of its initial value [199].

In another work, Wan *et al*. [212] used chitosan/bentonite and crosslinked chitosan immobilized on bentonite as adsorbent for Cu^2+^. Crosslinking agents are used to amend mechanical and chemical properties of the adsorbents.

Some researchers prepared nanocomposites using a natural carbohydrate (e.g., chitosan), a polymer and a layered compound [213–219]. Wang *et al*. [213] increased the number of compounds for synthesizing hybrid polymer and prepared chitosan-*g-*poly(acrylic acid)/attapulgite/sodium humate composite hydrogels for the adsorption of Pb^2+^. In this adsorbent, –NH_2_ groups of chitosan (CSA), –COOH and –COO^−^ groups of polyacrylic acid (PAA), –COO^−^ and Ph–CO^−^ groups of sodium humate (SH) and Si–OH groups of attapulgite (APT) adsorbed Pb^2+^ ions. Depending on the used quantity of the above constituents, the adsorption capacity of hybrid polymer for Pb^2+^ was in the range of 702.35 to 843.86 mg/g.

Chitosan-*g*-poly(acrylic acid)/attapulgite (CTS–*g*–PAA/APT) composites were used for the fast adsorption of Cu^2+^ [214]. Results showed that attapulgite (APT) really reacted with the CTS–*g*–PAA polymer (Supporting information Figure S5). Adsorption experiments were carried out at pH = 5.5. It was fast and more than 90% of Cu^2+^ was adsorbed after about 15 min. FTIR spectra of CTS–*g*–PAA/APT compound before and after the adsorption of Cu^2+^ showed that –NH_2_, –OH groups of chitosan and –COOH groups of PAA in the composites interacted with Cu^2+^. This compound had a high *q*_max_ (785.2 mg/g) for the adsorption of Hg^2+^ [215], as well.

Poly(methacrylic acid)-grafted chitosan/bentonite (CTS–g–PMAA/Bent) composite (Supporting information Figure S6) was synthesized for the adsorption of U(VI) [216]. They used *N*,*N’*-methylenebisacrylamide as a crosslinking agent. XRD patterns showed that bentonite was exfoliated during the formation of composite. The adsorption process was carried out at pH = 5.5 in which the CTS–g–PMAA/Bent surface charge was negative and UO_2_(OH)^+^ was the predominant species. XPS spectra showed that –COO^−^ groups of composite interacted with UO_2_^2+^. Similarly, methacrylic acid grafted chitosan/bentonite [217], Chitosan-*g*-poly(acrylic acid)/vermiculite [218] and humic acid-immobilized-amine modified polyacrylamide immobilized on bentonite [219] were used for the adsorbtion of a number of heavy metal cations.

**Figure 15.**
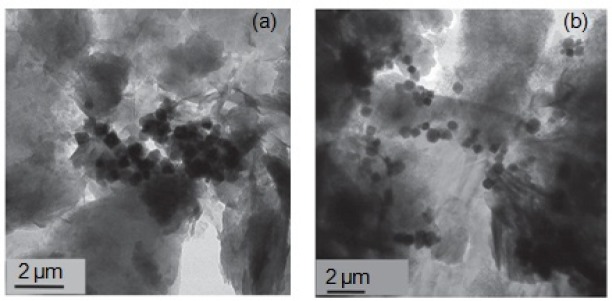
TEM images of (**a**) Fe_3_O_4_–PEI800–MMT and (**b**) Fe_3_O_4_–PEI2500–MMT hybrid materials. Reprinted with permission from [220]. Copyright 2012 Elsevier.

**Scheme 6.**
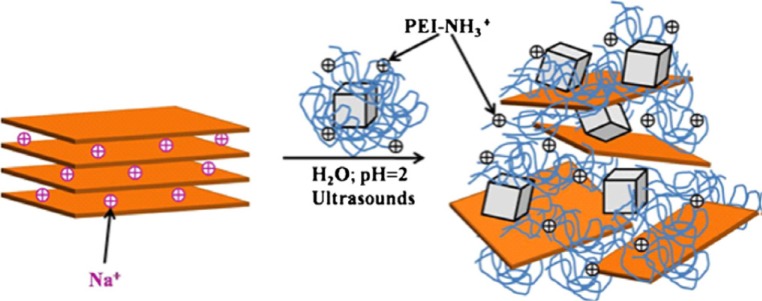
Schematic representation of Fe_3_O_4_–PEI*x*–MMT formation. Reprinted with permission from [220]. Copyright 2012 Elsevier.

In another groups of organically-functioalized layered compounds, synthetic polymers were used as organic moiety. Fe_3_O_4_ nanoparticles coated with polyethylenimine (PEI) polymer were intercalated between sodium rich montmorillonite (MMT) layers [220] under acidic conditions (pH = 2) and used it as a magnetic sorbent for the adsorption of Cr(VI). At pH = 2, amine groups of PEI were protonated and intercalated between MMT platelets by cationic exchange (Scheme 6). Two different molecular weights of PEI were investigated (*x* = 800 or 25,000 g/mol). The TEM images of Fe_3_O_4_–PEI*x*–MMT compound showed MMT existed as individual exfoliated platelets and intercalation tactoids composed by a few sheets (Figure 15).

A better dispersion of magnetites was obtained in Fe_3_O_4_–PEI2500–MMT. The pH of zero point of charge of magnetite was 8.3 and the amine groups of PEI were protonated at pH below 10.4 and adsorbed Cr(VI) polyanions (CrO_4_^2−^, HCrO_4_^−^ and Cr_2_O_7_^2−^) through electrostatic interactions. In pH lower than 2, Cr(VI) ions were mainly as H_2_CrO_4_ and the adsorption decreased. Experiments showed that they can be used for a wide pH range and the optimum pH for the adsorption process was 6. The *q*_max_ values of Fe_3_O_4_–PEI800–MMT and Fe_3_O_4_–PEI25000–MMT compounds were 8.77 and 7.69 mg/g, respectively.

**Scheme 7.**
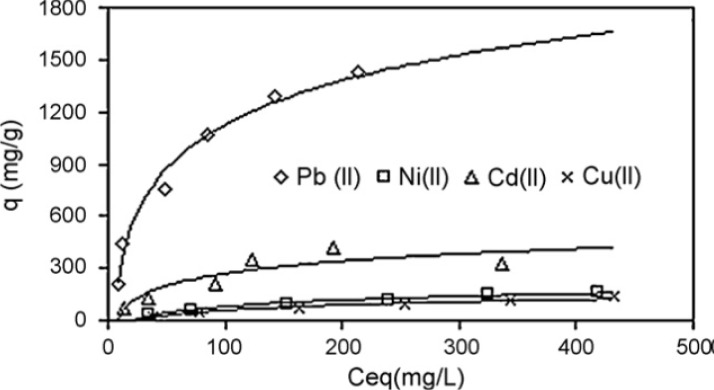
Adsorption isotherms of Pb^2+^, Ni^2+^, Cd^2+^ and Cu^2+^. Reprinted with permission from [221]. Copyright 2009 Elsevier.

A novel superabsorbent composite was synthesized by copolymerization reaction of partially neutralized acrylic acid (AA) on bentonite micropowder using *N*,*N*′-methylenebisacrylamide as a crosslinker [221]. The superabsorbent composite (SAC) was characterized by Fourier transform infrared spectroscopy (FTIR), thermogravimetric analysis (TGA) and scanning electron microscopy (SEM). The *q*_max_ values for adsorption of Pb^2+^, Ni^2+^, Cd^2+^ were 1666.67, 270.27, 416.67 and 222.22 mg/g respectively, Scheme 7.

Organic-modified montmorillonite coated by *N*-(4-vinylbenzyl)-*N*-methyl-*D*-glucamine polymer was studied in the presence of a crosslinking reagent for the adsorption of arsenate [222]. XRD patterns showed that the intercalation process was not complete. Also, TEM images confirmed the dispersion of montmorillonite within matrix. Adsorption experiments were carried out at pH = 6 and As(V) polyanions (as H_2_AsO_4_^−^) interacted with ammonium groups of adsorbent and its *q*_max_ was 72.99 mg/g.

**Figure 16.**
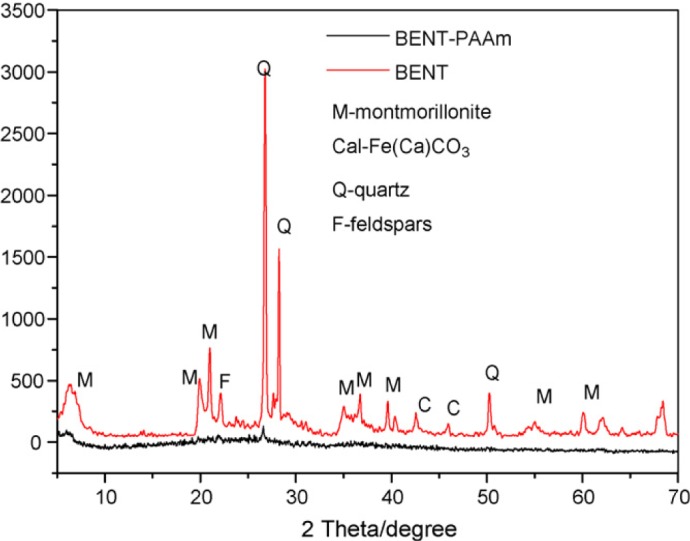
XRD patterns of Bentonite (BENT) and BENT–PAAm. Reprinted with permission from [223]. Copyright 2010 Elsevier.

Bentonite (BENT) embedded in polyacrylamide (PAAm) composite was used [223] for the removal of Cu^2+^ from water. XRD patterns of BENT and BENT–PAAm (Figure 16) shows that acrylamide polymerization destroyed BENT structure and resulted in the crystal confusion. Results showed that the optimum pH for the adsorption of Cu^2+^ was 6.2. Increasing pH, increased the negatively charged or deprotonated amines and SiOH groups of BENT–PAAm and also increased the hydrolysis of Cu(II).

Below pH = 6.5, Cu(II) is mainly as Cu^2+^ (Supporting information Figure S7). In pHs of 5 and 6.2, *q*_max_ values of BENT–PAAm were bigger than those of BENT which was due to the presence of amine groups of PAAm. On the other hand, it was reported that negatively charged sites of bentonite in BENT–PAAm composite adsorbed Pb^2+^ [224]. In this compound, polyacrylamide chains just increase thermal and mechanical stabilities of the nanocomposite and its *q*_max_ for the adsorption of Pb^2+^ is less than that of pure bentonite.

Polyaniline/attapulgite (PANI/ATP) composite was used achieving a high adsorption of mercury [225]. X-ray photoelectron spectroscopy showed that after the adsorption of Hg^2+^ on PANI/ATP, its amine (–NH–) content reduced from 6.97 to 4.55 atom% and its imine (–NH^+^–) content also diminished from 4.54 to 0.96 atom%. This showed that Hg^2+^ was adsorbed on both amine and imine functional groups. The optimum pH for the adsorption process was 6 and its *q*_max_ values at ionic strengths of 0.01, 0.1 and 1M were 909.1, 813.1 and 781.3 mg/g, respectively. As reported before [226], polyacrylamide/attapulgite adsorbed Hg^2+^ via its –NH_2_ functional groups and its *q*_max_ value was 192.5 mg/g. It was observed that Cl^−^ decreased drastically its adsorption capacity of Hg^2+^ via forming HgCl_4_^2−^ compound. The adsorption capacity of PANI/ATP was preserved at 93% by the fifth cycle. Also, as seen in Table 3, –OH, –SO_3_^−^ and –NH– groups of organic moiety in polyvinyl alcohol/attapulgite [227], acrylamide-2-acrylamido-sodium 2-methylpropane sulfonate/clay [228] and poly(methoxyethyl)acrylamide/clay [229], respectively had a relatively high affinity for adsorption of Pb^2+^.

### 3.4. Adsorption of Heavy Metals on Organic-Inorganic Core-Shell Nanocomposites (CSNs)

CSNs have different applications and were studied briefly in Section 2.3.4. Here, we discuss the role of these compounds in the adsorption of metal ions. A number of examples [233–252] have been given in Table 4.

**Scheme 8.**
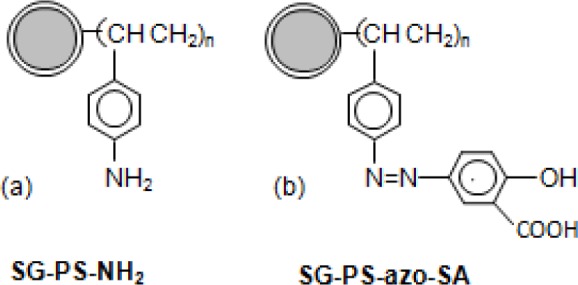
Structures of (**a**) SG–PS–NH_2_ and (**b**) SG–PS–azo–SA. Reprinted with permission from [233]. Copyright 2010 Springer Science and Business Media.

Silica gel is a commonly used supporting material in inorganic-organic hybrid materials due to its environmental and economic factors and high thermal and mechanical stabilities [233–241]. Polystyrene diazo-coupled with salicyclic acid [233] (SG–PS–azo–SA) and encapsulated silica gel by polystyrene containing amino groups (SG–PS–NH_2_) [234] (Scheme 8) were used for the removal of a number of heavy metals.

Thermogravimetric analysis of SG–PS–NH_2_ and SG–PS–azo–SA showed that the respective organic layer had a high thermal stability. Also, the values of BET surface area, pore volume and pore size decreased greatly from silica gel to SG–PS–NH_2_ to SG–PS–azo–SA. SEM images of silica gel, SG–PS–NH_2_ and SG–PS–azo–SA indicated that the spherical shape and sizes of these three samples were similar (Supporting information Figure S8), proving that the microspheres of silica gel had good mechanical stability during the process of reaction. The *q*_max_ values of SG–PS–NH_2_ for Cu^2+^, Ag^+^ and Au^3+^ were 0.17, 0.47 and 0.59 mmol/g [234] and those of SG–PS–azo–SA were 1.29, 1.85 and 1.61 mmol/g, respectively [233]. Amine groups of SG–PS–NH_2_ and –N=N– and salicyclic acid groups of SG–PS–azo–SA are their interacting groups.

On the other hand, adsorption capacities of organic functional groups change considerably depending on the nature of inorganic moiety. For example, *q*_max_ value of polyacrylamide in SiO_2_/polyacrylamide [237] for Hg^2+^ was much less than that of polyacrylamide/attapulgite [226].

**Figure 17.**
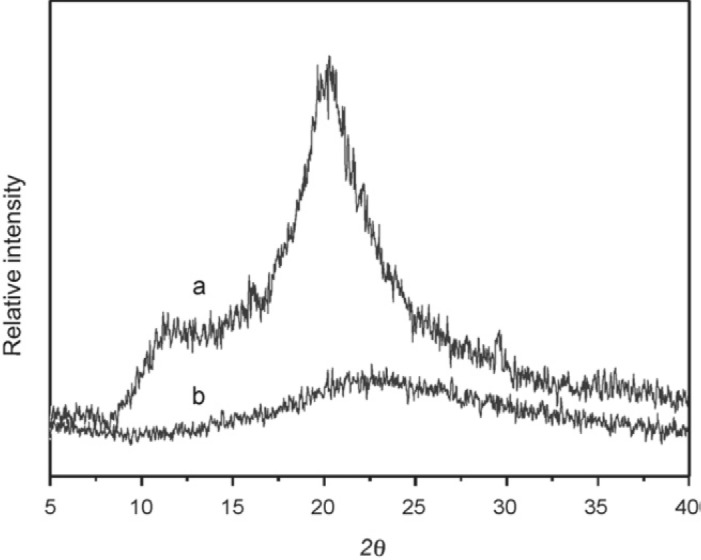
XRD patterns of (**a**) pure chitosan and (**b**) chitosan in non-supported hybrid material. Reprinted with permission from [239]. Copyright 2007 Elsevier.

Some authors have used natural carbohydrate and biomass as shell of their adsorbents. Immobilization of biomass (*Chetoceros* sp microalgae) on a SiO_2_ core resulted in an adsorbent for adsorption of Pb^2+^ [238]. Chitosan-grafted silica gel imprinted by sucrose and polyethylene glycol 4000 (PEG 4000) was used for the adsorption of Cu^2+^ [239]. This method has been used for direct preparation of porous sorbent with low mass transfer resistance, available functional ligand and excellent mechanical resistance [240]. Sucrose and PEG 4000 interacted with chitosan via hydrogen bonds and extraction of them, by breakage of H-bonds, resulted in a porous structure. The optimum pH for the adsorption of Cu^2+^ on this adsorbent was 6. At this pH, copper was mostly as Cu^2+^ form and most of amine groups of chitosan (its interacting group) were as –NH_2_. TG/DSC analyses showed that the thermal stability of grafted chitosan was higher than pure chitosan (Supporting information Figure S9). The XRD patterns show no peak for crystallization regions of chitosan in non-supported hybrid material compared to those of pure chitosan which might be ascribed to the demolition of its crystallinity (Figure 17). The *q*_max_ values of Cu^2+^ adsorption for the adsorbent imprinted with sucrose and PEG 4000, PEG 4000, sucrose and non-imprinted adsorbent [241] were 10.5, 9.1, 3.2 and 0.2 mmol/g, respectively.

**Scheme 9.**
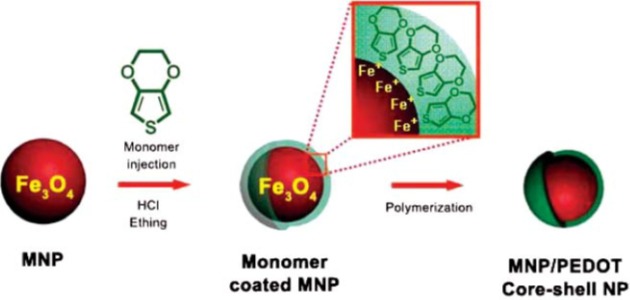
Synthesis steps of Fe_3_O_4_–PEDOT nanoparticles by seeded polymerization mediated with acidic etching. Reproduced with permission from [243]. Copyright 2007 The Royal Society of Chemistry.

The used adsorbent was washed by 0.1 M HCl and the capacity of the regenerated sorbent through five cycles was detected to be 94% of the fresh one and equilibration time in the case of sucrose and PEG 4000 was 25 min. Also, *q*_max_ value of SiO_2_(CO_2_H)/chitosan for adsorption of Ni^2+^ was higher than that of SiO_2_/chitosan [241] which shows effect of support on the adsorption property of adsorbent [242]. Another material that was used frequently in core-shell nanocomposites was Fe_3_O_4_ magnetic nanoparticle [243–248]. These kinds of compounds can be separated with the help of an external magnetic force. In a number of works, organic polymers were used as shell for Fe_3_O_4_ core [243,244]. Jang *et al*. [243] encapsulated Fe_3_O_4_ by poly(3,4-ethylenedioxythiophene) (PEDOT) and used the produced compound (Fe_3_O_4_–PEDOT) for the removal of metal ions (Scheme 9). PEDOT has an excellent environmental stability and can interact with positively charged metal ions through its sulfur atom. The PEDOT shell has a lower surface energy than the core of magnetic nanoparticles, thus Fe_3_O_4_–PEDOT nanoparticles show amended stability and dispersibility in aqueous solutions, compared to pristine Fe_3_O_4_ nanoparticles. Adsorption experiments showed that adsorption capacities of Fe_3_O_4_–PEDOT nanoparticles were: Ag^+^ > Hg^2+^ > Pb^2+^. It was regenerated by acid treatment and the recovered adsorbent by ten cycles showed no loss in its adsorption capacity.

Chitosan-bound Fe_3_O_4_ magnetic nanoparticle has been used for the adsorption of Cu^2+^ and Au(III) ions [245,246]. TEM images showed that Fe_3_O_4_ and chitosan-bound Fe_3_O_4_ nanoparticles had a similar particle size of 13.5 nm and were monodisperse [245]. This revealed the reaction between Fe_3_O_4_ and chitosan occured only on the surface of Fe_3_O_4_. Also, XRD patterns showed that the binding of chitosan to the surface of Fe_3_O_4_ did not result in the phase change of Fe_3_O_4_. Experiments showed that the pH of isoelectric point of chitosan-bound Fe_3_O_4_ nanoparticles was 5.9 and the optimum pH for the adsorption of Cu^2+^ ions was in the range of 2–5 [245] and that of Au(III) negatively-charged complex was 2 [246]. The *q*_max_ values for Cu^2+^ and Au(III) were 21.5 [245] and 59.52 mg/g [246], respectively. In a different study, Tao *et al*. [247] used thiol-functionalized magnetic mesoporous microsphere (TMMM) for adsorption of Hg^2+^ and Pb^2+^. The Fe_3_O_4_ nanoparticles were used as a core and coated with two silica layers, a tightly crossed silica thin layer and a mesoporous silica shell. The thiol moiety had a strong affinity towards these metal ions. The porous structure of the silica shell brought large surface area and provided the opportunity to graft organic moiety for adsorption of metal ions. *q*_max_ value of Hg^2+^ was 185.19 mg/g at 20 °C and decreased in the presence of Ca^2+^, Mg^2+^ and Na^+^ ions.

In some works, organic moieties (chitosan [248] and polystyrene [249]) were used as core. Polystyrene-supported Fe_3_O_4_ nanoparticles (PS–Fe_3_O_4_ NPs) was used for the adsorption of arsenate oxyanion [249]. Zeta potential measurements showed the polystyrene (PS) latex was negatively charged in a wide pH range and Fe_3_O_4_ nanoparticles were positively charged in acidic or neutral solutions. This was the driving force for the acervation of Fe_3_O_4_ nanoparticles on PS and the formation of PS-Fe_3_O_4_ NPs. At pH of 6, As(V) was as HAsO_4_^2−^ and PS–Fe_3_O_4_ NPs had the maximum adsorption capacity for its adsorption. TEM images showed that the diameter of fresh Fe_3_O_4_ spherical beads (due to aggregation) were in the range of 350–400 nm, but there was not observed coalescence between PS–Fe_3_O_4_ NPs. Due to these, the *q*_max_ value for the adsorption of arsenate on PS–Fe_3_O_4_ was 139.3 mg/g whereas that of Fe_3_O_4_ was 78.4 mg/g. The used PS-Fe_3_O_4_ NP was recovered by 0.1 M NaOH and its recycling efficiency after the sixth cycle was 89.6%.

Lv *et al*. [250] synthesized nanosized hydrous MnO_2_ encapsulated within porous polystyrene cation exchanger as adsorbent for heavy metals. The –SO_3_^−^ groups of polymeric matrix and –MnOH and –Mn(OH)_2_ groups of MnO_2_ interacted with Zn^2+^ and Cd^2+^. In the presence of Ca^2+^, due to its electrostatic interaction with –SO_3_^−^ groups, *q*_max_ values of nanocomposite for adsorption with Zn^2+^ and Cd^2+^ were decreased greatly.

**Scheme 10.**
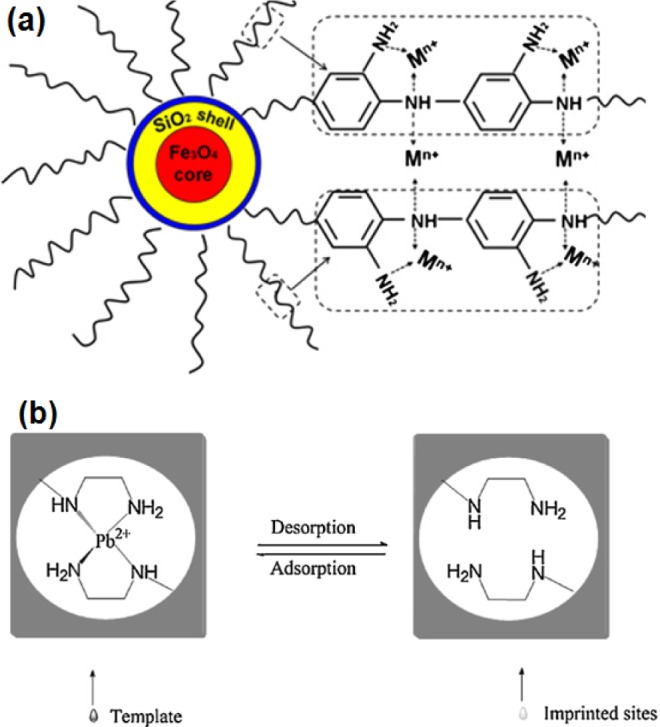
Schematic structures of (**a**) FSP (Reprinted with Permission from [251]. Copyright 2012 Elsevier) and (**b**) template in Fe_3_O_4_@SiO_2_@IIP (Reprinted with permission from [252]. Copyright 2011 Elsevier).

Some researchers have used two inorganic compounds (e.g., SiO_2_ and Fe_3_O_4_) as the core [251,252]. Firstly, they prepared Fe_3_O_4_ on which a layer of SiO_2_ was synthesized, and then carried out the polymerization on the surface of SiO_2_. Song *et al*. [251] synthesized Fe_3_O_4_–SiO_2_–poly(1,2-diaminobenzene) sub-micron particle (FSP) for the removal of Cr(III), As(III) and Cu(II). Zhang *et al*. [252] used 3-(2-aminoethylamino)propyltrimethoxysilane (AAPTS) as the functional monomer and Pb^2+^ as the template to prepare an ion-imprinted polymer (Fe_3_O_4_@SiO_2_@IIP) for the separation of Pb^2+^ ion from water (Scheme 10).

XRD patterns showed that peaks of Fe_3_O_4_ of the core-shell compounds were similar to those of naked Fe_3_O_4_ nanoparticles and the surface modification of Fe_3_O_4_ did not result in its phase change. In both adsorbents, amine groups interacted with metal ions. Experiments using Fe_3_O_4_@SiO_2_@IIP at pH = 7.5 showed that in the presence of Cu^2+^, Zn^2+^, Cd^2+^ and Hg^2+^, the relative selectivity factor values of Pb^2+^/Cu^2+^, Pb^2+^/Zn^2+^, Pb^2+^/Cd^2+^ and Pb^2+^/Hg^2+^ were 7.41, 6.76, 3.75 and 6.39, respectively [252]. However, when authors used a non-imprinted polymer to synthesize this core-shell polymer (Fe_3_O_4_@SiO_2_@NIP), values of the above-mentioned relative selectivity factor were about 1 and this adsorbent was not selective toward Pb^2+^.

As we saw in this section, synthesing core-shell nanocomposites does not result in the phase change of its inorganic moiety. In some cases, more than one inorganic compound was used as the core. Functional groups of the shell have a major role in the adsorption of heavy metal ions and core-shell nanocomposites had good mechanical stability and organic moiety of these compounds had higher thermal stability than that of its pure form.

### 3.5. Adsorption of Heavy Metals on Organic-Inorganic Hierarchically Structured Materials

**Figure 18.**
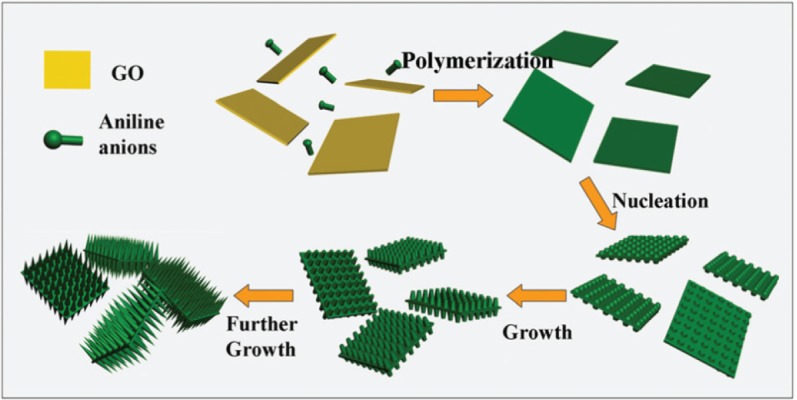
Mechanism of formation of polyaniline (PANI) nanorods on the surface of graphene oxide (GO) (PANI/GO) hierarchical nanocomposites. Reproduced with permission from [253]. Copyright 2013 The Royal Society of Chemistry.

Hierarchically structured compounds were explained briefly in Section 2.3.5. Li *et al*. [253] synthesized a hierarchical nanocomposite by the polymerization of aniline arrays on the surface of grapheme oxide nanosheets and used it as a superadsorbent for the adsorption of Cr(VI) oxyanion from water (Figure 18 and Table 4). Aniline monomers (ANI) were adsorbed on the surface of graphene oxide (GO) through electrostatic interactions with its reactive oxygen-containing functional groups and further the polymerization of ANIs resulted in the formation of aligned polyaniline (PANI) nanorods on the surface of GO (PANI/GO).

XRD patterns showed that the interplanar distance of GO nanosheets was 0.88 nm (as agglomerated) and PANI/GO demonstrated characteristic peaks of PANI (Supporting information Figure S10). These evidence proved that GO and PANI/GO were as agglomerated and separated nanosheets, respectively. Adsorption experiments were carried out at pH = 2 in which Cr(VI) adapting the form of HCrO_4_^−^ interacted with the protonated nitrogen atoms of PANI in pure PANI and PANI/GO. Pure PANI was simply aggregated and its surface area was smaller than PANI/GO and the *q*_max_ values of PANI and PANI/GO were 490.2 and 1149.4 mg/g, respectively.

**Figure 19.**
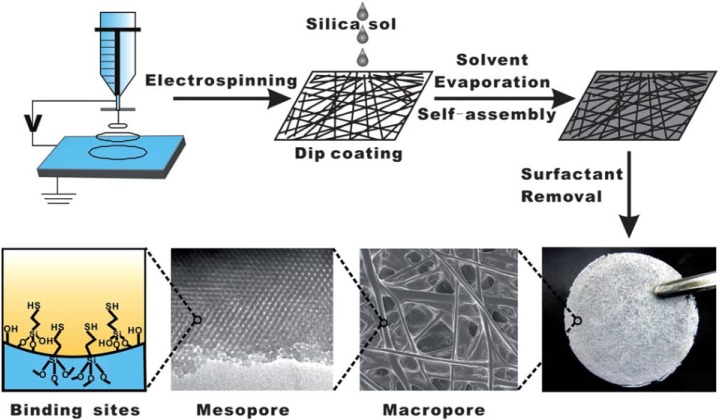
Schematic illustration of preparing thiol-functionalized membranes using electrospun nanofibrous mats as the skeleton. Reproduced with permission from [254]. Copyright 2012 The Royal Society of Chemistry.

In a novel work, Li *et al*. [254] fabricated polystyrene (PS) nanofibrous mats by an electrospinning method and used as a skeleton for thiol-functionalized mesoporous silica compound (Figure 19 and Table 4). In this hierarchical structure the PS nanofibers had macroporous structures (diameters of 3–10 μm) and were randomly distributed in the membrane (Supporting information Figure S11). The optimum pH for the adsorption of Cu^2+^ was about 5 and its *q*_max_ value was 11.33 mg/g. Cu^2+^ ions were adsorbed by –SH groups within mesopores. The amount of thiol groups in the adsorbent was 0.32 mmol/g and the adsorption efficiency (Cu^2+^/SH molar ratio) of adsorbent was about 80%. After the recovery of adsorbent by 0.5 M HCl, its adsorption capacity of Cu^2+^ was basically maintained after five cycles.

**Figure 20.**
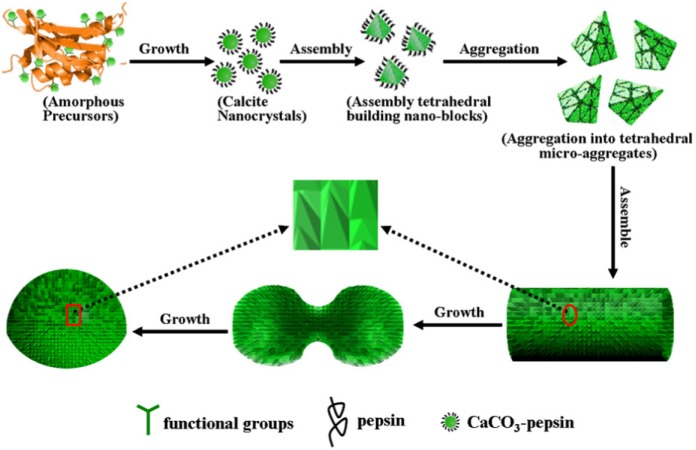
Mechanism of the formation of hierarchical structures of CaCO_3_–pepsin from amorpuus precursors. Reprinted with permission from [255]. Copyright 2012 Elsevier.

In another different works with hierarchical structure compounds, Ma *et al*. synthesized CaCO_3_–pepsin [255] and CaCO_3_–maltose [256] and used them as adsorbents. Here, structure and adsorption characteristics of CaCO_3_–pepsin [255] are studied (Figure 20). Pepsin is an enzyme which is made up of 327 amino acids. SEM images showed that CaCO_3_–pepsin consisted of a large number of tetrahedral micro-aggregates that were composed of smaller tetrahedral building nano-blocks (Supporting information Figure S12).

XRD, SEM and IR results showed that CaCO_3_ and pepsin interacted through both coordination of Ca^2+^ and pepsin and interactions between the surface of CaCO_3_ nanocrystals and –OH and –CO groups of pepsin.

Adsorption experiments were carried out in neutral pH and the adsorption capacity of CaCO_3_–pepsin for Pb^2+^ and Cu^2+^ were 1167 and 611 mg/g, respectively and the removal of Pb^2+^ was very rapid (about 20 min). Solubility product of PbCO_3_ and CuCO_3_ are less than CaCO_3_ and thus Cu^2+^ and Pb^2+^ precipitate as their carbonates.

On the other hand, adsorption capacities of CaCO_3_–maltose [256], another hierarchical structure superadsorbent, for Pb^2+^, Ni^2+^, Cu^2+^ and Cd^2+^ (as metal carbonate) were 3242.48, 769.23, 628.93 and 487.8 mg/g, respectively whereas adsorption capacity of CaCO_3_ for Pb^2+^ is 62.5 mg/g and this showed clearly synergic effects between organic and inorganic moieties in these compounds. As we saw, the organic-inorganic hierarchically structured compounds are superadsorbent.

### 3.6. Characteristics of Organic-Inorganic Hybrid Polymers

To study Sections 3.1–3.5, the characteristics of different kinds of organic-inorganic hybrid polymers were as follows: (1) Some functional groups of these adsorbents are –SH, –COOH, amines and –S–S–, –OH, –SO_3_^−^ and ion exchange sites of hybrid polymer; (2) adsorption processes mostly obey the Langmuir isotherm that shows the adsorption is monolayer and there is a uniform distribution of sites on the surface of adsorbents; (3) the adsorption capacity of each certain organic functional group changes depending on its content [181,214], the used organic compound, inorganic support [166,195], crosslinking agent [212], aggregation of nanoparticles [253] and the used synthesis method [182,189]; (4) in some cases, organic compounds are used only for increasing thermal and mechanical stability of adsorbents and without adsorbing heavy metal ions [224]; (5) mesoprous compounds, due to their porous structures have a rather higher adsorption capacities compared to those of organically-functionalized layered, core-shell compounds and products of sol-gel method; (6) heavy metals as cations and oxyanions are adsorbed via electrostatic interactions and the increase in the hydrophobicity of functional groups of adsorbent (e.g., –N(C_2_H_5_)_2_ [156] or from –NH_2_ to –NH(propyl) to –N(propyl)_2_ [178]) decreases their adsorption capacity for metal cations and increases their *q*_max_ values (e.g., from trimethylammonium to tri-*n*-butylammonium functional groups) for the adsorption of some hydrophobic oxyanions [183]; (7) In the most cases, for example [141–147,216,221,225,241,243,249,252], the used adsorbents are recovered by acid treatment and in the cases that acid dissolves the adsorbent [199], they have used another washing solutions; (8) some ions may react with metallic ions, e.g., Cl^−^ with Hg^2+^ [226], and inhibit their adsorption; (9) some ions, e.g., Cl^−^ [183], SO_4_^2−^ [196] or metallic cations [252], may react with functional groups of hybrid adsorbents and decrease their adsorption capacity for heavy metal ions; (10) steric hindrance on functional group decreases its adsorption capacity [151]; (11) in most cases, adsorption capacities of organic-inorganic hybrid polymers are bigger than those of their organic or inorganic constituents; (12) most of heavy metals were adsorbed in the pH range 4–7. With the increase in alkalinity of solution, heavy metal ions convert to metal hydroxides and this decreases their affinity for interaction with binding sites of adsorbents [224] and in highly acidic solutions, H^+^ ions compete with metal ions for adsorption on the adsorbent surface [212]; (13) increasing the content of functional groups increases the adsorption capacity of these kinds of hybrid polymers although it decreases their pore size and surface area [153,169,170]; and (14) adsorption capacities of hierarchically structure compounds are much greater than the other kinds of discussed organic-inorganic hybrid polymers and in the future they can be used as superadsorbents for the adsorption of heavy metals.

## 4. Conclusions and Outlook

Organic-inorganic hybrid polymers are obtained through sol-gel processes, self-assembly processes, assembling or dispersion of nanobuilding blocks, hierarchical structures and interpenetrating networks. In these compounds, the functional variation of organic materials combines with the benefits of a sturdy and thermally stable inorganic substrate. These materials have strong binding affinities toward selected metal ions (as cations or oxyanions) and relatively high metal ion adsorption capacities and can be used for wastewater treatment and solid-state separation of heavy metals. They are modified by suitable functional groups for high efficiency adsorption of heavy metal ions under the used experimental conditions. Techniques such as XRD and SEM show that in hybrid polymers, the structure of organic and inorganic moieties change and synergy effects between them increase their adsorption capacities, compared to their individual pristine organic or inorganic moieties. Heavy metal selective adsorbents can be prepared by methods such as cation-IIP. Although adsorbents similar to activated carbon are used routinely for their low cost and high adsorption capacity, different combinations of organic and inorganic moieties provide us a large number of selections of hybrid adsorbents that can be used under different experimental conditions, are reusable and can be prepared for selective adsorption of heavy metals and in some cases act as superadsorbents. For synthesis of these compounds, environmentally friendly compounds such as chitosan, humic acid, cellulose and bentonite are used and their partial leaching during adsorption processes has no negative impact on the environment.

## Supporting Information



## Figures and Tables

**Table 1.** Chemicals used for synthesizing adsorbents by sol-gel method, adsorbed heavy metals, maximum adsorption capacity, pH and temperature for adsorption of heavy metals from waters.

Chemicals (interacting group)	Heavy metal [*q*_max_, pH and *t* (°C)]	Isotherm	Reference
Pyromellitic acid dianhydride/phenylaminomethyl trimethoxysilane (–COOH)	Pb^2+^ (7.16 mmol/g, 5, room)	Langmuir	[142]
Pyromellitic acid dianhydride/phenylaminomethyl trimethoxysilane (–COOH)	Cu^2+^ (0.28 mmol/g, 4, room)	Langmuir	[143]
	Pb^2+^ (1.56 mmol/g, 5, room)	Langmuir	
3-Thiocyanatopropyltriethoxysilane/TEOS (–SH)	Cd^2+^ (87.7 mg/g, 5, 25)	Langmuir	[144]
3-[2-(2-Aminoethylamino)ethylamino]propyl-trimethoxysilane (Cd^2+^– imprinted hybrid sorbent) (–NH_2_)	Cd^2+^ (77.2 mg/g, 6, 25)	Langmuir	[145]
Cd^2+^-imprinted mercapto-functionalized silica gel (–SH)	Cd^2+^ (83.89 mg/g, 6, 27)	Experimental	[146]
Non-imprinted mercapto-functionalized silica gel (–SH)	Cd^2+^ (35.91 mg/g, 6, 27)	Experimental	–
Fe^3+^-imprinted cyanato-functionalized silica gel (–C≡N)	Fe^3+^ (35.6 mg/g, 3, 20)	Experimental	[147]
3-Glycidyloxypropyltrimethoxysilane/potassium tert-butoxide/titanium isopropoxide (oxygen atoms of adsorbent)	Pb^2+^ (181.2 mg/g, 5.5, room)	Langmuir	[148]
	Cu^2+^ (44.64 mg/g, 5.5, room)	Langmuir	
	Cd^2+^ (35.84 mg/g, 5.5, room)	Langmuir	
Zr(OCH_2_CH_2_CH_3_)_4_/3-mercapto-1-propanesulfonic acid (–SH)	Pb^2+^ (0.36 mmol/g, neutral, room)	Experimental	[149]
	Hg^2+^ (0.87 mmol/g, neutral, room)	Experimental	
Ti(OCH_2_CH_2_CH_3_)_4_/3-mercapto-1-propanesulfonic acid (–SH)	Pb^2+^ (1.24 mmol/g, neutral, room)	Experimental	–
	Hg^2+^ (1.41 mmol/g, neutral, room)	Experimental	
7-Amine-4-aza-heptyltrimethoxisilane/TEOS (amine group)	Pb^2+^ (36.64 mg/g, neutral, room)	Langmuir	[150]
10-Amine-4-aza-decyltrimethoxisilane/TEOS (amine group)	Pb^2+^ (30.27 mg/g, neutral, room)	Langmuir	–
3-Chloropropyltrimethoxysilane/TEOS (nitrogen center of sorbent)	Ni^2+^ (0.47 mmol/g, 4.5, room)	Langmuir	[151]
3-Aminopropyl triethoxysilane/TEOS (–NH_2_)	Pb^2+^ (45.45 mg/g, 6, 25)	Langmuir	[152]
	Cu^2+^ (35.71 mg/g, 6, 25)	Langmuir	–
3-Chloropropyltrimethoxysilane/TEOS (nitrogen center of sorbent)	Mn^2+^ (0.35 mmol/g, 4.5, room)	Langmuir	–
Amino-terminated dendrimer-like PAMAM polymer/silica gel (amino content 1.91 mmol/g) (–NH– and –NH_2_)	Cu^2+^ (78.7 mg/g, ethanolic, 25)	Langmuir	[153]
2-Aminoethyl-3-aminopropyltrimethoxysilane/poly(dimethyl siloxane) (–NH/NH_2_)	Cu^2+^ (1.2 mmol/g, ethanolic, 25)	Langmuir	[154]
	Ni^2+^ (0.37 mmol/g, ethanolic, 25)		
	Fe^3+^ (1.3 mmol/g, ethanolic, 25)		
Bis[3-(triethoxysilyl)propyl]disulfide/TEOS (–S–S–)	Cd^2+^ (26.8 mg/g, 6, 25)	Langmuir	[155]
	Pb^2+^ (56.7 mg/g, 6, 25)	Langmuir	
	Cu^2+^ (13.3 mg/g, 6, 25)	Langmuir	
	Salen(NEt_2_)_2_/silica gel (–OH and –N(C_2_H_5_)_2_)	Cu^2+^ (4.36 mg/g, 11, room)		Langmuir	[156]
Mn^2+^ (2.34 mg/g, 11, room)		Langmuir			
Pb^2+^ (10.06 mg/g, 11, rom)		Langmuir			
Zn^2+^ (11.3 mg/g, 11, room)	Langmuir
2-Acrylamido-2-methylpropanesulfonic acid/silica gel (–OH, –NH– and –SO_3_H)	Cu^2+^ ( 0.66 mmol/g, ethanolic, 25)	Langmuir	[157]
Amino-terminated dendrimer-like polyamidoamine polymer/silica gel (–NH_2_)	Pd^2+^ (0.7 mmol/g, neutral, room)	Experimental	[158]
	Pt^4+^ (0.41 mmol/g, neutral, room)	Experimental	
	Au^3+^ (0.12 mmol/g, neutral, room)	Experimental	
Polyethylene glycol/3-mercaptopropyl trimethoxysilane (–SH)	Cu^2+^ (0.4 mmol/g, 4, 40)	Langmuir	[159]
Polyvinilalcohol/3-(2-aminoethylamino)propyl trimethoxysilane (–NH/NH_2_)	Pb^2+^ (67.6 mg/g, 5, 30)	Langmuir	[160]
Activated alumina/3-mercaptopropyl trimethoxysilane (–SH)	As(III) (9.28 mg/g, 7, room)	Experimental	[161]
N-[3-(trimethoxysilyl)propyl]-ethylenediamine/TEOS (–NH_2_)	Pt^2+^ (139 mg/g, 3.05, 25)	Langmuir–Freundlich	[162]
*Escherichia coli*/TEOS (immobilized *E. coli* cells)	Cd^2+^ (79.9 mg/g, 5.7–6.2, 25)	Langmuir	[163]
N,N-(dipropylcarbamothioyl)thiophene-2-carboxamide/TEOS (–S–, C=O and amine groups)	Cd^2+^ (3.89 mg/g, 7, 30)	Langmuir	[164]
SiNSSH/TEOS (via sulfur or nitrogen atoms of sorbent)	Hg^2+^ (8.52 mmol/, neutral, 25)	Langmuir	[165]
	Pb^2+^ (1.90 mmol/g, neutral, 25)	Langmuir	
	Cu^2+^ (1.66 mmol/g, neutral, 25)	Langmuir	
	Ni^2+^ (1.44 mmol/g, neutral, 25)	Langmuir	
	Co^2+^ (1.26 mmol/g, neutral, 25)	Langmuir	
3-Aminopropyltriethoxysilane/TEOS (–NH_2_)	Ni^2+^ (31.29 mg/g, 4.5, 25)	Langmuir	[166]
	Cd^2+^ (40.73 mg/g, 4.5, 25)	Langmuir	
	Pb^2+^ (96.79 mg/g, 4.5, 25)	Langmuir	
3-Chloropropyltrimethoxysilane/aniline/TEOS (–NH–)	Co^2+^ (0.32 mmol/g, 4.5, 25)	Experimental	[167]
	Zn^2+^ (0.34 mmol/g, 4.5, 25)	Experimental	
	Cd^2+^ (0.12 mmol/g, 4.5, 25)	Experimental	

**Table 2.** Adsorbed heavy metals, maximum adsorption capacity, pH and temperature for adsorption of heavy metals by mesoporous compounds.

Adsorbent (interacting group)	Heavy metal (*q*_max_, pH and *t* (°C))	Isotherm	Reference
SBA-16 modified with n-propyl-salicylaldimine (–NH_2_)	Cu^2+^ (58 mg/g, 4, room)	Experimental	[168]
	Co^2+^ (16 mg/g, 4, room)	Experimental	
SBA-16 modified with n-propyl-salicylaldimine and salicylaldehyde (–N=)	Cu^2+^ (15.2 mg/g, 4, room)	Experimental	–
	Co^2+^ (4.5 mg/g, 4, room)	Experimental	
SiO_2_ modified with n-propyl-salicylaldimine (–NH_2_)	Cu^2+^ (45.2 mg/g, 4, room)	Experimental	–
	Co^2+^ (11.2 mg/g, 4, room)	Experimental	
SiO_2_ modified with n-propyl-salicylaldimine and salicylaldehyde (–N=)	Cu^2+^ (10.2 mg/g, 4, room)	Experimental	–
	Co^2+^ (3.6 mg/g, 4, room)	Experimental	
3-Aminopropyl-functionalized MCM-41 (ammonium group)	Arsenate (64.4 mg/g, 3–4,room)	Experimental	[169]
	Chromate (52.9 mg/g, 7–8,room)	Experimental	
3-Aminopropyl-functionalized SBA-1 (ammonium group)	Arsenate (94.2 mg/g, 3–4,room)	Experimental	–
	Chromate (132.7 mg/g, 7–8,room)	Experimental	
H_2_N–(CH_2_)_2_–NH–(CH_2_)_3_–functionalized SBA-15 (–NH_2_ and –NH–)	Cu^2+^ (0.83 mmol/g, neutral, 25)	Langmuir	[170]
3-Aminopropyl-functionalized SBA–15–N–C–H (–NH_2_)	Cu^2+^ (0.39 mmol/g, neutral, 25)	Langmuir	–
3-Aminopropyl-functionalized SBA–15–N–E (–NH_2_)	Cu^2+^ (0.35 mmol/g, neutral, 25)	Langmuir	–
3-Aminopropyl-functionalized SBA–15–N–C (–NH_2_)	Cu^2+^ (0.24 mmol/g, neutral, 25)	Langmuir	–
3-(2-Aminoethylamino) propyltrimethoxysilane modified ordered mesoporous silica	As(V) (10.3 mg/g, 7, room)	Experimental	[171]
N-propyl aniline-functionalized MCM-41 (–NH–)	Arsenate (0.85 mmol/g, 4.2, room)	Experimental	[172]
	Hg^2+^ (0.92 mmol/g, 3.5, room)	Experimental	
	Pb^2+^ (0.78 mmol/g, 5.8, room)	Experimental	
H_2_N–functionalized SBA-15 (–NH_2_)	Cu^2+^ (1.28 mg/g, neutral, room)	Experimental	[173]
	Pb^2+^ (1.31 mg/g, neutral, room)	Experimental	
	Cd^2+^ (1.35 mg/g, neutral, room)	Experimental	
Imidazole-functionalized SBA-15 (N atoms of imidazole)	Pd^2+^ (0.091 mmol/g, 4, room)	Experimental	[174]
	Pt^2+^ (0.091 mmol/g, 4, room)	Experimental	
SBA-15 modified with 3-aminopropyl-triethoxysilane and salicylaldehyde (–N= and –O^−^ groups)	Cu^2+^ (46 mg/g, 4.8, room)	Experimental	[175]
	Ni^2+^ (22 mg/g, 4.8, room)	Experimental	
	Co^2+^ (19 mg/g, 4.8, room)	Experimental	
	Zn^2+^ (26 mg/g, 4.8, room)	Experimental	
Amino-functionalized mesoporous silica (–NH_2_ and –OH)	Ni^2+^ (2.48 mmol/g, 8.5, 25)	Sips	[176]
2-Mercaptopyrimidine-functionalized SBA-15 (N and S atoms)	Cd^2+^ (0.99 mmol/g, 6, 25)	Experimental	[177]
3-Aminopropyl-functionalized MCM-41 (–NH_2_)	Au^3+^ (0.4 mmol/g, 2.5, room)	Experimental	[178]
NH(propyl)-functionalized MCM-41 (–NH–)	Au^3+^ (0.33 mmol/g, 2.5, room)	Experimental	–
N(propyl)_2_-functionalized MCM-41 (N atom of amine group)	Au^3+^ (0.2 mmol/g, 2.5, room)	Experimental	–
Chemically modified MCM-41 (–NH– and –NH_2_)	Hg^2+^ (0.7 mmol/g, 5, 25)	Langmuir	[179]
3-Aminopropyl-functionalized MCM-41 (–NH_2_)	Ag^+^ (0.62 mmol/g, 5, 22)	Experimental	[180]
	Cu^2+^ (0.84 mmol/g, 5, 22)	Experimental	
3-Mercaptopropyl-functionalized MCM-41 (–SH)	Ag^+^ (0.97 mmol/g, 5, 22)	Experimental	–
	Cu^2+^ (0.02 mmol/g, 5, 22)	Experimental	
3-Mercaptopropyl-functionalized SBA-16 (–SH)	Cu^2+^ (36.42 mg/g, 5.5, 25)	Langmuir	[181]
3-Aminopropyl-modified SBA-15 (–NH_2_)	Cu^2+^ (73.5 mg/g, 6.3, 25)	Langmuir	[182]
SBB or *N*-((trimethoxysilyl)propyl)-*N*,*N*,*N*-tri-*n*-butylammonium chloride-functionalized SBA-15 (quaternary ammonium)	ReO_4_^−^ (1.85 mmol/g, 6.4, room)	Experimental	[183]
3-Aminopropyl and 3-mercaptopropyl bi-functionalized mesoporous silica (–SH)	Hg^2+^ (1.51 mmol/g, neutral, room)	Experimental	[184]
Meso-structured silica modified with 3-mercaptopropyltrimethoxy silane and 9-(chloromethyl)anthracene (–SH)	Pb^2+^ (13.96 mg/g, neutral, 25)	Experimental	[185]
	Cu^2+^ (12.56 mg/g, neutral, 25)	Experimental	
	Hg^2+^ (12.09 mg/g, neutral, 25)	Experimental	
	Zn^2+^ (3.69 mg/g, neutral, 25)	Experimental	
3-Mercaptopropyl-functionalized SBA-15 (–SH)	Hg^2+^ (2.88 mmol/g, 4.5, 20)	Langmuir	[186]
Ordered mesoporous silica modified with 2,5-dimercapto-1,3,4-thiadiazole (–SH)	Hg^2+^ (1.7 g/g, neutral, room)	Experimental	[187]
1-Benzoyl-3-propylthiourea-functionalized MCM-41 (=N, =O, –NH– and –NH_2_ groups)	Hg^2+^ (1 g/g, neutral, room)	Experimental	[188]
Disulfide-bridged periodical mesoporous organosilica (–S–S–)	Hg^2+^ (716 mg/g, 2, room)	Experimental	[189]
3-Mercaptopropyl-functionalized MCM-41 (–SH)	Hg^2+^ (0.59 mmol/g, neutral, room)	Experimental	[36]
3-Mercaptopropyl-functionalized HMS (–SH)	Hg^2+^ (1.5 mmol/g, neutral, room)	Experimental	–
Mesoporous thioether-functionalized polyvinylpyrrolidone (PVP)/SiO_2_ composite (–S–)	Hg^2+^ (4.26 mmol/g, 2, room)	Experimental	[190]
Disulfide-functionalized SBA-1 (–SH)	Hg^2+^ (849 mg/g, 2, room)	Experimental	[191]
3-(((5-ethoxybenzenethiol)imino)methyl)-salicylic acid immobilized onto mesoporous silica (–N= and –SH groups)	Pd^2+^ (164.2 mg/g, 3, room)	Langmuir	[192]
3-(3-(Methoxycarbonyl)benzylidene) hydrazinyl)benzoic acid immobilized onto mesoporous silica (C=O and –N=NH–)	Cu^2+^ (145.98 mg/g, 7, room)	Langmuir	[193]
3-(2-Aminoethylamino)propyl-functionalized mesoporous silica (–NH– and –NH_2_)	Cu^2+^ (0.107 mmol/g, 3, room)	Experimental	[194]
3-Aminopropyl-functionalized MCM-41 (–NH_2_)	Cd^2+^ (0.71 mmol/g, 5, 22)	Experimental	[195]
	Ni^2+^ (0.69 mmol/g, 5, 22)	Experimental	
Diamino-functionalized MCM-41	Co^2+^ (0.69 mmol/g, neutral, 25)	Experimental	[196]
	Ni^2+^ (0.52 mmol/g, neutral, 25)	Experimental	
Diamino-functionalized MCM-48	Co^2+^ (1 mmol/g, neutral, 25)	Experimental	–
	Ni^2+^ (0.86 mmol/g, neutral, 25)	Experimental	
Aminopropyl grafted SBA-15 modified with EDTA (–NH– and –COO^−^)	Pb^2+^ (273.2 mg/g, 5, 25)	Langmuir	[197]
CONH_2_-functionalized SBA-15 (CONH_2_ group)	Cu^2+^ (1.4 mmol/g, 5, 298)	Langmuir	[198]

**Table 3.** Adsorbed heavy metals, maximum adsorption capacity, pH and temperature for adsorption of heavy metals by organic polymer/layered compound hybrids.

Adsorbent (interacting group)	Heavy metal (*q*_max_, pH and *t* (°C))	Isotherm	Reference
Chitosan/cloisite 10 A (protonated –NH_2_)	Cr(VI) (357.14 mg/g, 3, 35)	Langmuir	[199]
Chitosan/bentonite (ion exchange property of bentonite and –OH and –NH_2_ groups of chitosan)	Pb^2+^ (15 mg/g, 4, 25)	Langmuir	[200]
	Cu^2+^ (12.6 mg/g, 4, 25)	Langmuir	
	Ni^2+^ (6.1 mg/g, 4, 25)	Langmuir	
Chitosan/perlite (protonated –NH_2_)	Cr(VI) (153.8 mg/g, 4, 25)	Experimental	[201]
Chitosan/perlite (–NH_2_ and –OH)	Cu^2+^ (196.08 mg/g, 5, room)	Langmuir	[202]
	Ni^2+^ (114.95 mg/g, 5, room)	Langmuir	
Chitosan/perlite (–NH_2_)	Cd^2+^ (178.6 mg/g, 6, 25)	Experimental	[203]
Chitosan/perlite (–NH_2_ and –OH)	Cu^2+^ (1.59 mmol/g, 4.5, 25)	Langmuir	[204]
Chitosan/clinoptilolite (–NH_2_)	Cu^2+^ (11.32 mmol/g, 5, 25)	Langmuir	[205]
	Co^2+^ (7.94 mmol/g, 5, 25)	Langmuir	
	Ni^2+^ (4.21 mmol/g, 5, 25)	Langmuir	
Chitosan/alumina (protonated amino groups)	Cr(VI) (9.71 mg/g, 4, 40)	Langmuir	[206]
Chitosan/alumina (protonated –NH_2_)	Cr(VI) (153.85 mg/g, 4, 25)	Langmuir	[207]
Chitosan/montmorillonite (protonated –NH_2_)	Cr(VI) (40.65 mg/g, 4, 35)	Langmuir	[208]
Chitosan/montmorillonite (protonated –NH_2_ group of chitosan)	Selenate (18.4 mg/g, 4, room)	Langmuir	[209]
Chitosan/calcium alginate (–NH_2_ and –OH)	Ni^2+^ (222.2 mg/g, 5, room)	Langmuir	[210]
Cellulose/hydroxyapatite (ion exchange property of hydroxyapatite)	Pb^2+^ (16.32 mg/g, neutral, 25)	Langmuir	[211]
Chitosan/bentonite (–NH_2_)	Cu^2+^ (9.85 mg/g, 4, 25)	Langmuir	[212]
Epichlorohydrin-crosslinked chitosan/bentonite (–NH_2_)	Cu^2+^ (11.75 mg/g, 4, 25)	Langmuir	–
Ethylene glycol diglycidyl ether-crosslinked chitosan/bentonite (–NH_2_)	Cu^2+^ (10.52 mg/g, 4, 25)	Langmuir	–
Glutaraldehyde-crosslinked chitosan/bentonite (–NH_2_)	Cu^2+^ (4.17 mg/g, 4, 25)	Langmuir	–
Chitosan-*g*-poly(acrylic acid)/attapulgite/sodium humate (–COO^−^, –COOH, –NH_2_ and –O^−^)	Pb^2+^ (809.5 mg/g, 5.5, 35)	Langmuir	[213]
Chitosan-*g*-poly(acrylic acid)/attapulgite (–COO^−^)	Cu^2+^ (303.03 mg/g, 5.5, 30)	Langmuir	[214]
Chitosan-*g*-poly(acrylic acid)/attapulgite (–COOH, –NH_2_ and –OH)	Hg^2+^ (785.2 mg/g, 5, 30)	Langmuir	[215]
Poly(methacrylic acid) grafted chitosan/bentonite (–COOH)	U(VI) (117.2 mg/g, 5.5, 30)	Langmuir	[216]
Poly-methacrylic acid grafted chitosan/bentonite (amino and hydroxyl groups of chitosan)	Hg^2+^ (125 mg/g, 6, room)	Langmuir	[217]
	Pb^2+^ (111 mg/g, 6, room)	Langmuir	
	Cd^2+^ (83 mg/g, 6, room)	Langmuir	
Chitosan-*g*-poly(acrylic acid)/vermiculite (–COOH, –NH_2_ and –OH)	Pb^2+^ (3.08 mmol/g, neutral, 30)	Langmuir	[218]
	Cd^2+^ (2.94 mmol/g, neutral, 30)	Langmuir	
Humic acid-immobilized-amine modified polyacrylamide/bentonite (–COOH)	Cu^2+^ (106.2 mg/g, 5, 30)	Langmuir	[219]
	Zn^2+^ (96.1 mg/g, 9, 30)	Langmuir	
	Co^2+^ (52.9 mg/g, 8, 30)	Langmuir	
Polyethylenimine800/magnetite-montmorillonite (protonated –NH_2_)	Cr(VI) (8.77 mg/g, 3, 25)	Langmuir	[220]
Polyacrylic acid crossliked by N,N′-methylenebisacrylamide/montmorillonite (–CONH– and –COOH)	Ni^2+^ (270.3 mg/g, neutral, 25)	Langmuir	[221]
	Pb^2+^ (1666.7 mg/g, neutral, 25)	Langmuir	
Poly[N-(4-vinylbenzyl)-N-methyl-D-glucamine]/montmorillonite (protonated tertiary amine)	As(V) (72.26 mg/g, 6, 30)	Langmuir	[222]
	As(V) (72.99 mg/g, 6, 40)	Langmuir	
	As(V) (82.64 mg/g, 6, 50)	Langmuir	
Polyacrylamide/bentonite (–NH_2_)	Cu^2+^ (32.81 mg/g, 6.2, 20)	Langmuir	[223]
Polyacrylamide**-**bentonite composite (negatively charged sites of bentonite)	Pb^2+^ (0.16 mmol/g, 4.5–5, room)	Langmuir	[224]
Phytic acid-modified polyacrylamide**-**bentonite composite (negatively charged sites of bentonite)	Pb^2+^ (0.18 mmol/g, 4.5–5, room)	Langmuir	–
Polyaniline/attapulgite (–NH– and –N=)	Hg^2+^ (909.1 mg/g, 6, 25)	Langmuir	[225]
Polyacrylamide/attapulgite (–NH_2_)	Hg^2+^ (192.5 mg/g, 4.4, 30)	Langmuir	[226]
Polyvinyl alcohol/attapulgite (–OH)	Pb^2+^ (169.5 mg/g, 5, 30)	Langmuir	[227]
Acrylamide-2-acrylamido-sodium 2-methylpropane sulfonate copolymer/clay (–SO_3_^−^)	Cu^2+^ (1.07 mmol/g, 4.5, 25)	Experimental	[228]
	Cd^2+^ (1.28 mmol/g, 4.5, 25)	Experimental	
	Pb^2+^ (1.03 mmol/g, 4.5, 25)	Experimental	
Poly(methoxyethyl)acrylamide/clay (–NH–)	Pb^2+^ (0.385 mmol/g, 5, 30)	Langmuir	[229]
3-Aminopropyltriethoxysilane/sepiolite (–NH_2_)	Fe^2+^ (0.44 mmol/g, 3, 25)	Langmuir	[230]
	Cu^2+^ (0.14 mmol/g, 4, 25)	Langmuir	
Triethoxy-3-(2-imidazolin-1-yl)propylsilane/sepiolite (negatively charged sites of sepiolite and N atoms of imidazolin)	Mn^2+^ (0.085 mmol/g, 4, 25)	Langmuir	[231]
	Cu^2+^ (0.13 mmol/g, 4, 25)	Langmuir	
	Fe^3+^ (0.05 mmol/g, 2.5, 25)	Langmuir	
	Zn^2+^ (0.035 mmol/g, 4, 25)	Langmuir	
	Co^2+^ (0.28 mmol/g, 4, 25)	Langmuir	
	Cd^2+^ (0.09 mmol/g, 4, 25)	Langmuir	
Iodine-modified chitosan/bentonite (I_2_ and I^−^)	Gas-phase Hg^0^ at 110 °C	Experimental	[232]

**Table 4.** Adsorbed heavy metals, maximum adsorption capacity, pH and temperature for adsorption of heavy metals by organic-inorganic core/shell and hierarchically structured nanocomposites.

Adsorbent (interacting group)	Heavy metal (*q*_max_, pH and *t* (°C))	Isotherm	Reference
Core/shell nanocomposites			
SiO_2_/salicyclic acid functionalized polystyrene (O atom of –COOH and N atom of –N=N–)	Cu^2+^ (1.29 mmol/g, 5, room)	Langmuir	[233]
	Ag^+^ (1.85 mmol/g, 5, room)	Langmuir	
	Au^3+^ (1.61 mmol/g, 2.7, room)	Langmuir	
SiO_2_/amino functionalized polystyrene (–NH_2_)	Cu^2+^ (0.17 mmol/g, neutral, room)	Experimental	[234]
	Ag^+^ (0.47 mmol/g, neutral, room)	Experimental	
	Au^3+^ (0.59 mmol/g, neutral, room)	Experimental	
SiO_2_/imidazole-functionalized polystyrene (–N=N– and imidazole)	Au^3+^ (1.7 mmol/g, 2.7, room)	Langmuir	[235]
SiO_2_/aniline formaldehyde condensate (–NH_2_)	Cu^2+^ (76.33 mg/g, 5.4–5.7, room)	Langmuir	[236]
SiO_2_/polyacrylamide (–NH_2_)	Hg^2+^ (26.5 mg/g, acidic, 40)	Experimental	[237]
SiO_2_/*Chetoceros* sp microalgae	Pb^2+^ (0.19 mmol/g, 5, 27)	Langmuir	[238]
SiO_2_/chitosan imprinted by sucrose (amino groups)	Cu^2+^ (3.2 mg/g, 6, 25)	Langmuir	[239]
SiO_2_/chitosan imprinted by polyethylene glycol 4000 (amino groups)	Cu^2+^ (9.1 mg/g, 6, 25)	Langmuir	–
SiO_2_/chitosan imprinted by sucrose and polyethylene glycol 4000 (amino groups)	Cu^2+^ (10.5 mg/g, 6, 25)	Langmuir	–
SiO_2_/Cd^2+^-imprinted chitosan (amino groups)	Cd^2+^ (1.14 mmol/g, 6, room)	Experimental	[240]
SiO_2_/chitosan (amino groups)	Cd^2+^ (0.58 mmol/g, 6, room)	–	–
SiO_2_/chitosan (–NH_2_ and –OH)	Cu^2+^ (0.2 mmol/g, 5.5, 25)	Experimental	[241]
SiO_2_/chitosan (amino groups)	Ni^2+^ (182 mg/g, 7, 25)	Langmuir	[242]
SiO_2_(CO_2_H)/chitosan (amino groups)	Ni^2+^ (210 mg/g, 7, 25)	Langmuir	–
Fe_3_O_4_/poly(3,4-ethylenedioxythiophene) (–O–)	Ag^+^ (27.96 mmol/g, neutral, room)	Experimental	[243]
	Hg^2+^ (16.02 mmol/g, neutral, room)	Experimental	
	Pb^2+^ (14.99 mmol/g, neutral, room)	Experimental	
γ-Fe_2_O_3_/polyrhodanine (oxygen, nitrogen and sulfur atoms of polyrhodanine)	Hg^2+^ (179 mg/g, 4, 25)	Langmuir	[244]
Fe_3_O_4_ nanoparticle/chitosan (amino groups)	Cu^2+^ (21.5 mg/g, 5, 27)	Langmuir	[245]
Fe_3_O_4_ nanoparticle/chitosan (amino groups)	Au^3+^ (59.52 mg/g, 2, 25)	Langmuir	[246]
Fe_3_O_4_ nanoparticle/thiol-functionalized mesoporous microsphere (–SH)	Hg^2+^ (185.19 mg/g, 5, 20)	Langmuir	[247]
	Pb^2+^ (114.7 mg/g, 5, 20)	Langmuir	
Chitosan/zerovalent iron nanoparticles (complexation between Fe and Arsenic)	As^3+^ (94 mg/g, 7, 25)	Langmuir	[248]
	Arsenate (119 mg/g, 7, 25)	Langmuir	
Polystyrene/nano-Fe_3_O_4_ (Fe_3_O_4_)	Arsenate (139.3 mg/g, 6, 25)	Langmuir	[249]
Nanosized hydrous MnO_2_/porous polystyrene cation exchanger resin (–SO_3_^−^, –Mn(OH) and –Mn(OH)_2_ groups)	Cd^2+^ (1.96 mmol/g, 4.7, 25)	Langmuir	[250]
	Zn^2+^ (1.67 mmol/g, 4.7, 25)	Langmuir	
Fe_3_O_4_/SiO_2_/poly(1,2-diaminobenzene) (–NH_2_, –NH– and –N=)	As^3+^ (84.5 mg/g, 6, 25)	Langmuir	[251]
	Cr^3+^ (77 mg/g, 5.3, 25)	Langmuir	
	Cu^2+^ (65 mg/g, 6, 25)	Langmuir	
SiO_2_/Fe_3_O_4_/ion-imprinted polymer (–NH –CH_2_ –CH_2_ –NH_2_)	Pb^2+^ (19.61 mg/g, 7.5, room)	Langmuir	[252]
SiO_2_/Fe_3_O_4_/non-imprinted polymer (–NH –CH_2_ –CH_2_ –NH_2_)	Pb^2+^ (6.57 mg/g, 7.5, room)	Experimental	–
Polyaniline nanorods on graphene oxide nanosheets (amine group)	Cr(VI) (1149.4 mg/g, 3, 25)	Langmuir	[253]
Silica/polystyrene (–SH)	Cu^2+^ (11.33 mg/g, 5, 15)	Langmuir	[254]
CaCO_3_-pepsin (CaCO_3_ and adsorption occurs through formation of PbCO_3_ and CuCO_3_)	Pb^2+^ (1167 mg/g, neutral, room)	Experimental	[255]
	Cu^2+^ (611 mg/g, neutral, room)	Experimental	
CaCO_3_-maltose (CaCO_3_ and adsorption occurs through formation of PbCO_3_, CuCO_3_, NiCO_3_ and CdCO_3_)	Pb^2+^ (3242.48 mg/g, 7, 25)	Langmuir	[256]
	Cd^2+^ (487.8 mg/g, 7, 25)	Langmuir	
	Cu^2+^ (628.93 mg/g, 7, 25)	Langmuir	
	Ni^2+^ (769.23 mg/g, 7, 25)	Langmuir	
