# Supplementary material for: Organic-Inorganic Hybrid Polymers as Adsorbents for Removal of Heavy Metal Ions from Solutions: A Review
Source: Materials (Basel). 2014 Jan 27;7(2):673–726. doi: 10.3390/ma7020673 (PMC5453072; doi:10.3390/ma7020673)

Supporting Information

**Figure S1.** View of the two types of cages in the MOF structure made by reaction of Zr_6_O_4_(OH)_4_(OMc)_12_ clusters with muconic acid. Reproduced with permission from [104]. Copyright 2010 The Royal Society of Chemistry.

**Figure S2.** Illustration for the preparation of a macro-mesoporous TiO_2_-graphene composite film. Reprinted with permission from [132]. Copyright 2011 American Chemical Society.

**Figure S3.** Schematic representation of (**a**) Pen and (**b**) its interacting group. Reprinted with permission from [153]. Copyright 2012 Elsevier.


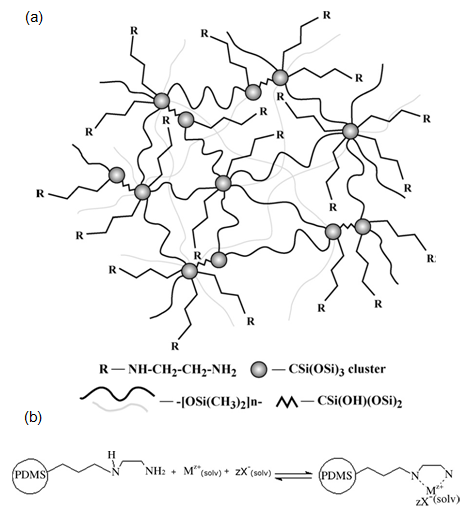


**Figure S4.** Adsorption of Cd^2+^ (◊) and Ni^2+^ (□) from their binary mixtures at (**a**) pH = 5 and (**b**) pH = 2. Reprinted with permission from [195]. Copyright 2007 American Chemical Society.

**
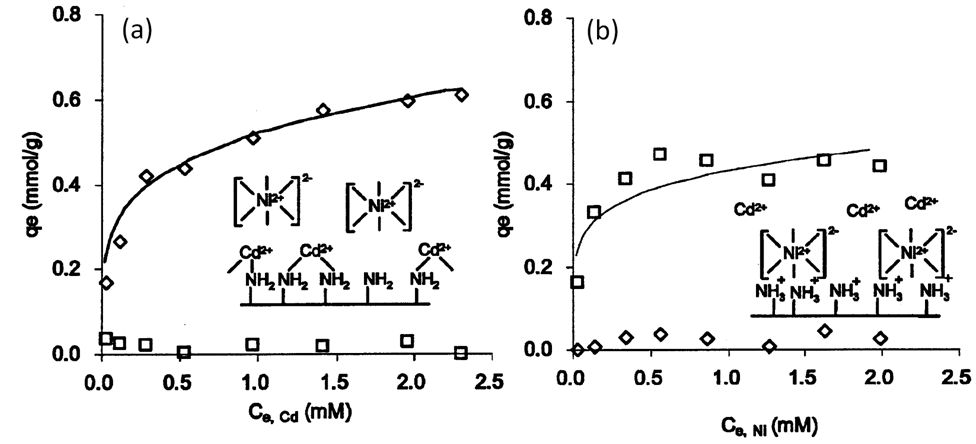
**

**Figure S5.** Schematic structure of CTS-*g*-PAA/APT composite. Reprinted with permission from [214]. Copyright 2009 Elsevier.

**Figure S6.** Schematic structure of of CTS-g-PMAA/Bent. Reprinted with permission
from [216]. Copyright 2012 Elsevier.

**Figure S7.** The distribution of different species of Cu(II) at concentration of 6.0 × 10^−4^ M in 0.01M NaClO_4_ and CO_2_-free solutions at 25 °C. Reprinted with permission from [224]. Copyright 2005 Elsevier.

**Figure S8.** SEM images of (**a**) silica gel; (**b**) SG–PS–NH_2_ and (**c**) SG–PS–azo–SA. Reprinted with permission from [233]. Copyright 2010 Springer Science and Business Media.


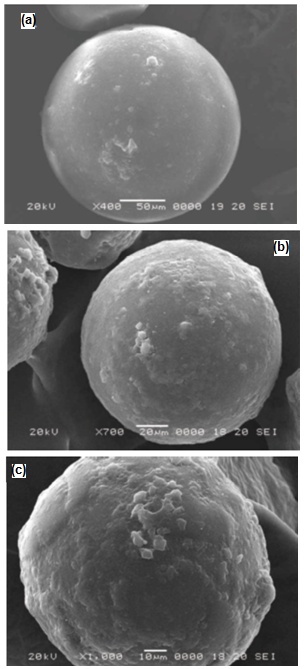


**Figure S9.** DSC analysis of (**a**) CS; (**b**) silica gel and (**c**) chitosan-grafted silica gel. Reprinted with permission from [239]. Copyright 2007 Elsevier.

**Figure S10.** XRD patterns of GO nanosheets, PANI nanorods, PANI/GO
nanocomposites. Reproduced with permission from [253]. Copyright 2013 The Royal Society of Chemistry.


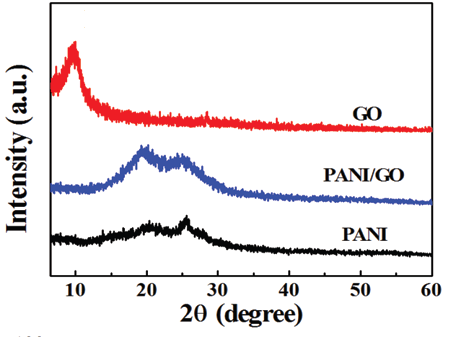


**Figure S11.** Pore size distributions of prepared membranes with bimodal porous
structure. Reproduced with permission from [254]. Copyright 2012 The Royal Society
of Chemistry.

**
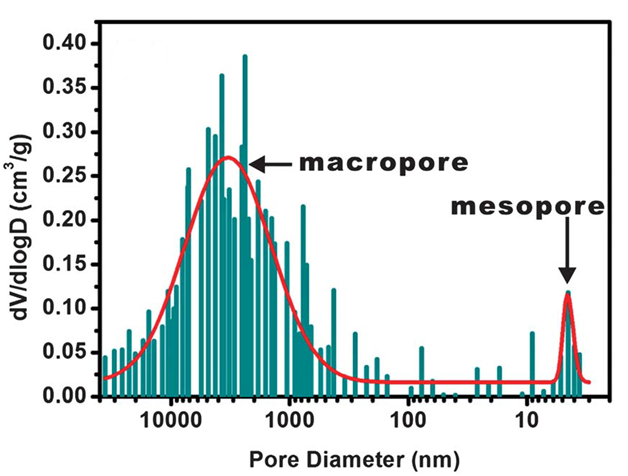
**

**Figure S12.** SEM images of CaCO_3_ crystals in pepsin aqueous solution (**a** and **b**). Reprinted with permission from [255]. Copyright 2012 Elsevier.


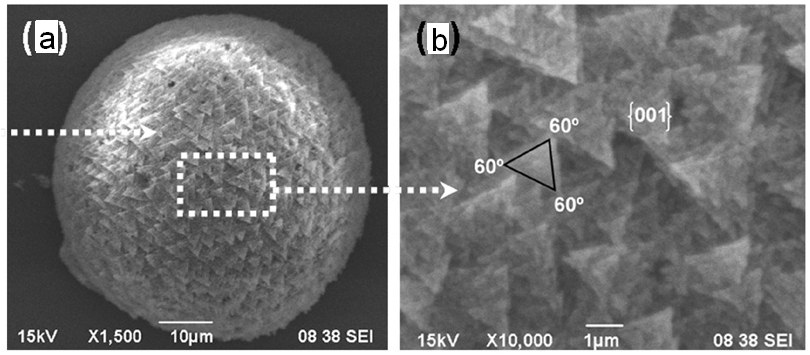

Supplement: Supplementary file 1 [file materials-07-00673-s001.docx]
